# Brevican, Neurocan, Tenascin-C, and Tenascin-R Act as Important Regulators of the Interplay Between Perineuronal Nets, Synaptic Integrity, Inhibitory Interneurons, and Otx2

**DOI:** 10.3389/fcell.2022.886527

**Published:** 2022-06-02

**Authors:** Cornelius Mueller-Buehl, Jacqueline Reinhard, Lars Roll, Verian Bader, Konstanze F. Winklhofer, Andreas Faissner

**Affiliations:** ^1^ Department of Cell Morphology and Molecular Neurobiology, Faculty of Biology and Biotechnology, Ruhr University Bochum, Bochum, Germany; ^2^ Department of Molecular Cell Biology, Institute of Biochemistry and Pathobiochemistry, Ruhr University Bochum, Bochum, Germany; ^3^ Department of Biochemistry of Neurodegenerative Diseases, Institute of Biochemistry and Pathobiochemistry, Ruhr University Bochum, Bochum, Germany; ^4^ Cluster of Excellence RESOLV, Ruhr University Bochum, Bochum, Germany

**Keywords:** brevican, neurocan, Otx2, parvalbumin, perineuronal nets, synapses, tenascin-C, tenascin-R

## Abstract

Fast-spiking parvalbumin interneurons are critical for the function of mature cortical inhibitory circuits. Most of these neurons are enwrapped by a specialized extracellular matrix (ECM) structure called perineuronal net (PNN), which can regulate their synaptic input. In this study, we investigated the relationship between PNNs, parvalbumin interneurons, and synaptic distribution on these cells in the adult primary visual cortex (V1) of quadruple knockout mice deficient for the ECM molecules brevican, neurocan, tenascin-C, and tenascin-R. We used super-resolution structured illumination microscopy (SIM) to analyze PNN structure and associated synapses. In addition, we examined parvalbumin and calretinin interneuron populations. We observed a reduction in the number of PNN-enwrapped cells and clear disorganization of the PNN structure in the quadruple knockout V1. This was accompanied by an imbalance of inhibitory and excitatory synapses with a reduction of inhibitory and an increase of excitatory synaptic elements along the PNNs. Furthermore, the number of parvalbumin interneurons was reduced in the quadruple knockout, while calretinin interneurons, which do not wear PNNs, did not display differences in number. Interestingly, we found the transcription factor Otx2 homeoprotein positive cell population also reduced. Otx2 is crucial for parvalbumin interneuron and PNN maturation, and a positive feedback loop between these parameters has been described. Collectively, these data indicate an important role of brevican, neurocan, tenascin-C, and tenascin-R in regulating the interplay between PNNs, inhibitory interneurons, synaptic distribution, and Otx2 in the V1.

## Introduction

The extracellular matrix (ECM) is an assembly of extracellular molecules secreted into the cellular microenvironment (Hynes and Naba, 2012). It can provide structural support to tissues through the formation of a meshwork consisting of protein–protein and protein–proteoglycan interactions (Yue, 2014). Furthermore, ECM components can serve as ligands for receptors and transmit signals involved in cellular processes such as proliferation, migration, apoptosis, and differentiation (Wang and Passaniti, 1999; Hynes, 2009; Padhi and Nain, 2020). Although the biochemical composition of the ECM is tissue-specific, the main components are collagens, proteoglycans, and glycoproteins (Murphy and Rudge, 1985; Frantz et al., 2010; Maeda, 2015). In the central nervous system (CNS), a specialized ECM called perineuronal net (PNN) can occur around certain neuron populations. Even though the discovery of PNNs by Camillo Golgi took place in the late 19th century (Celio et al., 1998), their important roles in cortical plasticity, regulation of neural activity, and association with neurodegenerative diseases began to emerge only recently (Wang and Fawcett, 2012; Dzyubenko et al., 2016; Lemarchant et al., 2016; Devienne et al., 2021). PNNs enwrap primarily inhibitory gamma-aminobutyric acid (GABA)ergic interneurons by forming a net-like structure around the soma and proximal dendrites (Lensjo et al., 2017). This arrangement of PNN components around the cell is often described as a honeycomb-shaped ECM structure with holes for synaptic contact. The largest population of neurons enwrapped by PNNs in the brain consists of parvalbumin-expressing neurons (Hartig et al., 1992; Devienne et al., 2021). PNNs show considerable molecular heterogeneity in terms of their composition (Miyata et al., 2018), but the main components of a basic general PNN structure consist of a hyaluronan backbone to attach the ECM to the cell surface (Deepa et al., 2006), the chondroitin sulfate proteoglycans (CSPGs) aggrecan, brevican, neurocan, and versican, which belong to the lectican family (Yamaguchi, 2000), and link proteins. Members of the hyaluronan and proteoglycan link protein (HAPLN) family can connect the lecticans to hyaluronan (Bekku et al., 2012). The trimeric glycoprotein tenascin-R (Tnr) can cross-link lectican CSPGs *via* the C-terminal G3-domain (Lundell et al., 2004; Morawski et al., 2014).

The emergence of PNNs around subsets of neurons is important for synaptic homeostasis. They restrict synaptic plasticity by maintaining synaptic stability, supporting the ability of neurons to adapt their synapses to inhibitory or excitatory signals, and play a key role in synaptogenesis (Happel and Frischknecht, 2016; Bozzelli et al., 2018; Fawcett et al., 2019). In the visual cortex, the maturation of parvalbumin-positive interneurons occurs during the onset of the critical period (Lu et al., 2014). The critical period is a brief well-defined phase, in which sensory input has a major impact on the wiring of local neuronal circuits and synaptic connections (Levelt and Hubener, 2012). The maturation of parvalbumin interneurons is accompanied by the condensation of the ECM as PNNs around these cells (Takesian and Hensch, 2013). It has been proposed that PNNs support the closure of plasticity by synapse stabilization and prevention of synaptic rearrangements and undertake this task even in adulthood (Pizzorusso et al., 2002). In addition, plasticity can be reopened even after the critical period by disrupting the PNNs (Pizzorusso et al., 2002). In rodents, reopening plasticity through PNN digestion can cure amblyopia, indicating the importance of PNNs in visual processing (Pizzorusso et al., 2006). This implies the key role of PNNs in the arrangement of inhibitory circuits and their synaptic integrity in the visual cortex as a response to visual sensory input. Another important component of parvalbumin interneuron maturation is the orthodenticle homeobox 2 (Otx2) transcription factor. Otx2 is transferred to the visual cortex from extracortical sources, including the retina and lateral geniculate nucleus, in an experience-dependent manner during postnatal development and also persists into adulthood (Sugiyama et al., 2008; Bernard and Prochiantz, 2016). The internalization of Otx2 by parvalbumin interneurons occurs after the binding of the protein to PNNs. When Otx2 binding is disturbed by PNN-degradation or specific inhibitors, the amount of Otx2 in parvalbumin interneurons is reduced (Beurdeley et al., 2012). In addition, a lower internalization of Otx2 by parvalbumin interneurons results in a reduced parvalbumin expression and PNN assembly (Beurdeley et al., 2012). A positive feedback loop between Otx2 and PNNs has been reviewed (Bernard and Prochiantz, 2016).

In the present study, we investigated the interplay between PNN components, synaptic stability, inhibitory interneurons, and Otx2 in the primary visual cortex (V1) of quadruple knockout mice. The quadruple knockout mouse lacks the four crucial ECM molecules brevican, neurocan, tenascin-C (Tnc) and Tnr (Rauch et al., 2005). This mouse line was generated because previously generated single knockout lines of brevican, neurocan, and both tenascins showed no major anatomical deficits (Forsberg et al., 1996; Weber et al., 1999; Zhou et al., 2001; Brakebusch et al., 2002). Tnr single knockout mice displayed an unchanged density of parvalbumin-positive cells in the somatosensory cortex, retrosplenial cortex, and the CA1 field of the hippocampus compared to wildtype mice (Weber et al., 1999). In contrast, Tnc deficient mice showed a significantly lower density of parvalbumin-positive cells in the cortex (Irintchev et al., 2005). Brevican regulates simultaneously cellular and synaptic forms of plasticity in parvalbumin-positive cells by modulating the localization of potassium channels and α-amino-3-hydroxy-5-methyl-4-isoxazolepropionic acid (AMPA) receptors (Favuzzi et al., 2017). However, to the best of our knowledge, investigations in brevican single knockouts on parvalbumin-positive cell density have not been performed yet. In addition, no alterations in the number of parvalbumin-positive cells in neurocan knockout animals are described. Single knockouts of the other lecticans, versican, and aggrecan, were embryonically and perinatally lethal (Watanabe et al., 1994; Mjaatvedt et al., 1998). Since the quadruple knockout appeared to be viable and fertile, it turned out to be a suitable model to study the flexibility of the ECM and the effects of the loss of four crucial ECM molecules and their missing interactions. An important feature of these four molecules is a strong connection to PNN structures. Brevican, neurocan, and Tnr are direct components of PNNs, and Tnc has also been associated with PNNs as a ligand for a variety of CSPGs (Galtrey et al., 2008; Zimmermann and Dours-Zimmermann, 2008; Morawski et al., 2014; Stamenkovic et al., 2017; Jakovljevic et al., 2021). Previous studies by our department showed a diminished PNN structure of quadruple knockout hippocampal neurons *in vitro*, accompanied by an impairment of synapse formation and stability. Synaptic activity in these cells was also disturbed, as shown by reduced miniature inhibitory postsynaptic currents (mIPSCs) and miniature excitatory postsynaptic currents (mEPSCs) (Geissler et al., 2013). Furthermore, an increase in excitatory synaptic elements and a reduction in inhibitory synaptic elements were observed in hippocampal quadruple knockout cultures, accompanied by an enhancement of neuronal network activity (Gottschling et al., 2019). To gain a deeper insight into the influence of the ECM on synaptic stability, inhibitory interneurons, and Otx2, we examined the visual cortex of quadruple knockout mice in this regard. We analyzed the number of PNN-wearing cells and parvalbumin and calretinin interneurons in cortical regions of wildtype control and quadruple knockout mice, highlighting the V1. Also, using structured illumination microscopy (SIM), high-resolution analyses of the impact of the quadruple knockout on the PNN structure were performed. In addition, the distribution of synaptic elements along the PNNs was investigated. Moreover, the occurrence of Otx2 in the V1 and the retrosplenial cortex (RSC) of wildtype and quadruple knockout mice was examined.

## Materials and Methods

### Animals

The mice were kept in the animal facility (Faculty of Biology and Biotechnology, Ruhr University Bochum) under a 12-hour (h) light–dark cycle with free access to chow and water. For the experiments, male and female 129/Sv wildtype (129S2/SvPasCrl; background mouse strain) and quadruple knockout mice (Rauch et al., 2005) were used at 14–16 weeks of age.

### Immunohistochemistry

Brains of male and female littermates of 14–16 week-old 129/Sv wildtype and quadruple knockout mice were fixed in 4% paraformaldehyde (PFA), cryoprotected, and embedded in Tissue-Tek freezing medium (Thermo Fisher Scientific, Cheshire, United Kingdom). For cell counting and basic quantification, brain tissue was sectioned coronally (16 μm; (interaural: 1.10-mm, bregma: −2.70-mm) using a cryostat (CM3050 S, Leica). Sections were blocked in a blocking solution containing 3% (v/v) normal goat serum (Dianova, Hamburg, Germany), 1% w/v bovine serum albumin (BSA; Sigma-Aldrich), and 0.5% Triton-X-100 (Sigma-Aldrich) in 1× PBS (phosphate-buffered saline) for 1 h at room temperature. Primary antibodies (Table 1) were diluted in blocking solution for 24 h. The sections were then washed three times for 10 min (min) with 1x PBS. Afterward, appropriate secondary antibodies were added and incubated for 2 h. Cell nuclei were detected using TO-PRO-3 (1:400; Thermo Fisher Scientific). To analyze the PNN structure and to detect synaptic proteins, free-floating sections (40 µm) were used. For the free-floating staining procedure, tissue sections were incubated in 1× PBS for 20 min and then blocked with a blocking solution containing 10% (v/v) normal goat serum (Dianova, Hamburg, Germany), 1% w/v BSA, and 0.1% (v/v) Triton-X-100 in 1× PBS for 1 h at room temperature. Primary antibodies were diluted in blocking solution and incubated at 4°C for 3 days. Next, tissue sections were washed three times with 1× PBS for 30 min and incubated with adequate secondary antibodies for 2 h.

**TABLE 1:** Antibodies for immunohistochemical stainings.

Primary antibody/Lectin	Species, clonality/type	Staining procedure	Dilution	Source/Research Resource Identifier (RRID)	Secondary antibody	Species	Dilution/source
Aggrecan	Rabbit, polyclonal, IgG	Glass slides	1:300	Merck KGaA; AB_90460	Anti-rabbit Cy5/Anti-rabbit Cy3	Goat	1:400 Dianova
Brevican	Mouse, monoclonal, IgG1	Free floating	1:200	BD Bioscience AB_398211	Anti-mouse Cy2	Goat	1:400 Dianova
Calretinin	Chicken, polyclonal, IgY	Glass slides	1:200	Synaptic Systems GmbH; AB_2619909	Anti-chicken Cy2	Goat	1:400 Dianova
Gephyrin	Mouse, monoclonal, IgG1	Free floating	1:200	Synaptic Systems GmbH; AB_887717	Anti-mouse Cy2	Goat	1:400 Dianova
Neurocan	Sheep, polyclonal, IgG	Free floating	1:200	R&D; AB_2149717	Anti-sheep Cy2	Donkey	1:400 Dianova
Otx2	Goat, polyclonal, IgG	Free floating	1:200	R&D; AB_2157172	Anti-goat Cy2	Donkey	1:400 Dianova
Parvalbumin	Chicken, polyclonal, IgY	Glass slides	1:100	Synaptic Systems GmbH; AB_2619887	Anti-chicken Cy2	Goat	1:400 Dianova
PSD95	Mouse, monoclonal, IgG2a	Free floating	1:300	Merck KGaA; AB_1121285	Anti-mouse Cy2	Goat	1:400 Dianova
Tenascin-C	Rabbit, polyclonal, IgG	Free floating	1:200	Faissner and Kruse (1990)	Anti-rabbit Cy3	Goat	1:400 Dianova
Tenascin-R (23-14)	Mouse, monoclonal, IgG	Free floating	1:200	Rathjen et al. (1991)	Anti-mouse Cy2	Goat	1:400 Dianova
VGLUT1	Guinea pig, IgG, polyclonal	Free floating	1:300	Synaptic Systems GmbH; AB_887878	Anti-guinea pig Cy5	Goat	1:400 Dianova
VGAT	Guinea pig, polyclonal, IgG	Free floating	1:300	Synaptic Systems GmbH; AB_887873	Anti-guinea pig Cy5	Goat	1:400 Dianova
*Wisteria floribunda* agglutinin	Lectin	Free floating/Glass slides	1:200	Vector Laboratories; AB_2336874	Streptavidin Cy3 Cy2		1:400 Dianova

### Fluorescence Stereo Microscopy and Confocal Laser Scanning Microscopy

For counting aggrecan, calretinin, parvalbumin, and WFA (*Wisteria floribunda* agglutinin) positive cells, coronal cortex sections were recorded using a fluorescence stereomicroscope (Axio Zoom.V16, Zeiss, Göttingen, Germany). 1.12 mm × 895.11 µm large areas of the V1 were selected in the left and right cortical hemispheres. The number of positive cells was then counted using ImageJ software (ImageJ 1.51w, National Institutes of Health; Bethesda, MD, United States). The overall aggrecan immunoreactive area [%] was analyzed as previously described (Reinehr et al., 2016). Therefore, images were converted into gray scale and background subtraction was performed with a rolling radius = 30. Next, lower and upper threshold values were determined for each image. The mean (lower threshold = 5.43 and upper threshold = 70.26) was used to analyze the percentage of the area fraction coherent. Otx2 staining in the RSC and V1 was examined using a confocal laser scanning microscope (LSM 510 META; Zeiss, Göttingen, Germany). Two images per animal (×630 magnification) were taken. As an indicator of PNN mediated Otx2 internalization, intensity measurements of WFA and Otx2 immunoreactivity in PNNs were performed using the CellProfiler 4.2.1 software (Stirling et al., 2021). We generated an automated analysis pipeline for the quantification of the signals. WFA-positive PNNs were the primary and the Otx2 signal within the PNN was the secondary identified object. For proper identification of WFA-positive PNNs, images had to be smoothed using a Gaussian filter. Because of the heterogeneity of the PNNs, the artifact diameter and smoothing scale thresholds had to be determined for each image individually. Next, mean intensities of the WFA and Otx2 signal for every single PNN were automatically measured. Intensity values were then normalized to the mean intensity value of all investigated wildtype RSC PNNs or all wildtype V1 PNNs. Measurements were performed at 79 PNN-enwrapped cells in the wildtype RSC and 71 in wildtype V1. In the quadruple knockout, 62 PNN-enwrapped cells in the RSC and 51 in V1 were analyzed. All the values were transferred to Statistica software (V13.3; StatSoft (Europe), Hamburg, Germany).

### Visualization of PNN Structure

To analyze the PNN structure in wildtype and knockout mice, immunohistochemical stainings of WFA were recorded using fluorescence super-resolution structured illumination microscopy (SIM) on a Zeiss Elyra PS.1 plus LSM880 microscope (Carl Zeiss Microscopy GmbH, Germany). Z-stack imaging with a ×63 oil immersion objective (Plan-Apochromat ×63/NA 1.4 OIL DIC, Carl Zeiss Microscopy GmbH, Germany) was used to acquire a stack of 60 sections with an interval of 0.3 μm. During all measurements, laser power and gain were kept constant. For 3D surface rendering and quantitative analysis of PNN parameters, SIM images were imported into IMARIS 9.3.1 (Bitplane AG, Zurich, Switzerland). First, the “*create surface*” tool was used to manually draw a surface containing the WFA-positive PNN for each optical section. An appropriate threshold was chosen to exclude the background signal. A 3D surface containing a single PNN-enwrapped neuron was generated and defined as a region of interest (ROI). WFA positive signal outside the ROI was suppressed. The volume of the generated ROI reflects the volume of the PNN. To determine the density of the PNN, a 3D surface of the WFA positive signal inside the ROI was generated, and its volume was automatically measured. By dividing the isolated WFA positive volume by the overall PNN volume, the amount of WFA positive signal around the neuron was identified and represented in percentage. In addition, the WFA total fluorescence intensity of every PNN was determined *via* IMARIS, and the intensity values were then normalized to the mean intensity value of the wildtype. For each group, 32 PNN-enwrapped cells were analyzed.

### Visualization of PNN-Associated Synaptic Puncta

The organization of inhibitory and excitatory synapses along PNNs in wildtype and knockout V1 was analyzed. Thus, immunohistochemical stainings either with WFA and antibodies against VGAT and gephyrin or WFA and antibodies against VGLUT1 and PSD95 were performed. Microscopy and generation of 3D surfaces of WFA positive PNNs were accomplished as described in Visualization of PNN Structure. In this ROI, the pre- and postsynaptic puncta distribution at the PNN was analyzed. Therefore, the “spots” analysis tool from the IMARIS software was used. To avoid the background signal from being detected as a synaptic component, a minimum diameter for synaptic puncta had to be defined. The diameters were determined by measuring the smallest synaptic puncta with the line tool in “slice view.” This resulted in 0.28 µm as the diameter for presynaptic markers (VGAT and VGLUT1) and 0.3 µm for postsynaptic markers (gephyrin and PSD95). Further noise was cancelled with the IMARIS software “background subtraction” tool. Hereby, settings were maintained for all examined PNNs. Next, the localization of the spots was identified. “Colocalize spots” was selected, and pre- and postsynaptic markers which were within a threshold distance of 1 µm of each other were labeled as colocalized. We used 1 µm as the distance between pre- and postsynaptic puncta to define them as colocalized, derived from other studies determining pre-and postsynapse in a spatial distance between 0.75 and 2.5 µm (Tamas et al., 2000; Schatzle et al., 2012; Fogarty et al., 2013). Spots outside the proximity of 1 µm were labeled as non-colocalized. Again, the synaptic distribution on 32 PNN-enwrapped cells was analyzed for the wildtype and quadruple knockout group.

### RNA Purification, cDNA Synthesis, and RT-qPCR

Primary visual cortex tissue was dissected and stored at −80°C (*n* = 7). RNA was isolated using the Gene Elute Mammalian Total RNA Miniprep Kit according to the manufacturer’s protocol (Sigma–Aldrich, St. Louis, MO, United States). The concentration of the isolated RNA was determined photometrically using a BioSpectrometer^®^ (Eppendorf, Hamburg, Germany). cDNA synthesis was performed using a cDNA synthesis kit (ThermoFisher Scientific, Waltham, MA, United States). Therefore, 1 µg RNA was reverse-transcribed with random hexamer primers. For quantitative real-time PCR (RT-qPCR) analyses, the Light Cycler 96^®^ System and SYBR Green I (Roche Applied Science, Mannheim, Germany) were used. The efficiency of the primer pairs (Table 2) was determined *via* a dilution series of 5, 25, and 125 ng cDNA. For normalization, the housekeeping gene *β-actin* was used.

**TABLE 2:** Primer sequences for RT-qPCR. bp = base pairs, for = forward, rev = reverse.

Gene	Primer sequence	Amplicon size (bp)	GenBank accession number
*Actb (β-actin)*	For: 5′-CTA​AGG​CCA​ACC​GTG​AAA​AG-3′	104	NM_007393.5
	Rev: 5′-ACC​AGA​GGC​ATA​CAG​GGA​CA-3′		
*Acan (aggrecan)*	For: 5′-CCA​GCC​TAC​ACC​CCA​GTG-3′	66	NM_007424.3
	Rev: 5′-GAG​GGT​GGG​AAG​CCA​TGT-3′		
*Calb2 (calbindin 2/ calretinin)*	For: 5′-CGA​AGA​GAA​TTT​CCT​TTT​GTG​C-3′	82	NM_001368293.1
	Rev: 5′-TGT​GTC​ATA​CTT​CCG​CCA​AG-3′		
*Dlg4 (PSD-95)*	For: 5′-TCT​GTG​CGA​GAG​GTA​GCA​GA-3′	110	NM_007864.3
	Rev: 5′-CGG​ATG​AAG​ATG​GCG​ATA​G-3′		
*Slc17a7 (VGlut1)*	For: 5′-GCA​GGA​GGA​GTT​TCG​GAA​G-3′	103	NM_182993.2
	Rev: 5′-GTC​GGC​ACT​CAG​CTC​CAG-3′		
*Slc32a1 (VGAT)*	For: 5′-ACG​TGA​CAA​ATG​CCA​TTC​AG-3′	84	NM_009508.2
	Rev: 5′-TGA​GGA​ACA​ACC​CCA​GGT​AG-3′		
*Gphn (gephyrin)*	For: 5′-TGG​TCT​CA0TCA​GTT​ATT​CCC​ATC-3′	72	NM_145965.2
	Rev: 5′-CGA​GAA​ATG​ATG​GAG​TCT​GGA-3′		
*Pvalb (parvalbumin)*	For: 5′-ACA​CTG​CAG​CGC​TGG​TCA​TA-3′	91	NM_001330686.1
	Rev: 5′-CCT​GCA​ACT​GTT​TGA​GCG​GG-3′		

### Western Blot Analyses

V1 tissue (*N* = 8/group) was homogenized in 100 μl lysis buffer (60 mM n-octyl-β-D-glucopyranoside, 50 mM sodium acetate, 50 mM tris chloride, pH 8.0, and 2 M urea) supplemented with protease inhibitor cocktail (Sigma-Aldrich) on ice for 1 h. Afterward, the samples were centrifuged at 14.000 × *g* at 4°C for 30 min. Then, the protein concentration in the supernatant was determined using a BCA protein assay kit (Pierce, Thermo Fisher Scientific, Rockford, IL, United States). To each protein sample (20 µg), 4× sodium dodecyl sulfate (SDS) was added. Next, samples were denaturized at 94°C for 5 min. Proteins were then separated *via* SDS-PAGE (10% and 15% polyacrylamide gels or 4-12% polyacrylamide gradient gels). Subsequently, proteins were transferred to a polyvinylidene difluoride (PVDF) membrane (Roth, Karlsruhe, Germany) by Western blotting (1–2 h and 75 mA). Blocking of the membranes was achieved with milk powder (5% w/v milk powder in 1× Tris-buffered saline (1× TBS). Additionally, membranes were incubated with primary antibody (Table 3) in a blocking solution overnight at 4°C. The following day, membranes were washed three times for 10 min with 1× TBST (1× TBS with 0.05% Tween^®^20). Incubation with horseradish peroxidase (HRP) coupled secondary antibody (Table 3) in blocking solution for 1 h was accomplished at room temperature. Membranes were washed three times with 1× TBST and two times with 1× TBS. For signal detection, ECL substrate solution (Bio-Rad Laboratories GmbH, München, Germany) was applied to the membrane and immunoreactivity was recorded with a MicroChemi Chemiluminescence Reader (Biostep, Burkhardtsdorf, Germany). For the evaluation of the protein signals, band intensity was analyzed using ImageJ software and normalized to a corresponding reference protein (actin). Actin staining was performed on the same membranes and lanes after incubation of the antibody of interest. In some cases, the membrane was stripped before actin was applied. Wildtype and quadruple knockout samples were always analyzed on the same membrane in an alternating manner. Normalized values of the Western blot results were given in arbitrary units (a.u.).

**TABLE 3:** List of primary and secondary antibodies for Western blotting.

Primary antibody	Species, clonality, type	Dilution	Source/RRID	Secondary antibody, type	Dilution	Source	kDa
Actin	Mouse, monoclonal, IgG	1:5000	BD Bioscience; AB_399901	Anti-mouse, IgG + IgM HRP	1:5000	Dianova	42
Aggrecan	Rabbit, polyclonal, IgG	1:10000	Merck KGaA; AB_ 90460	Anti-rabbit, IgG HRP	1:5000	Dianova	150
Calretinin	Chicken, polyclonal, IgY	1:1000	Synaptic Systems GmbH; AB_2619909	Anti-chicken, IgG HRP	1:5000	Dianova	31
Gephyrin	Mouse, monoclonal. IgG1	1:5000	Synaptic Systems GmbH; AB_887717	Anti-mouse, IgG + IgM HRP	1:5000	Dianova	93
Parvalbumin	Chicken, polyclonal, IgY	1:5000	Proteintech Germany GmbH; AB_2880541	Anti-chicken, IgG HRP	1:5000	Dianova	12
PSD95	Mouse, monoclonal, IgG2a	1:1000	Merck KGaA; AB_1121285	Anti-mouse, IgG + IgM HRP	1:5000	Dianova	95
VGAT	Guinea pig, polyclonal, IgG	1:2000	Synaptic Systems GmbH; AB_887873	Anti-guinea pig, IgG HRP	1:5000	Dianova	46
VGLUT1	Guinea pig, polyclonal, IgG	1:1000	Synaptic Systems GmbH; RRID: AB_887878	Anti-guinea pig, IgG HRP	1:5000	Dianova	62
Vinculin	Mouse, monoclonal, IgG1	1:200	Sigma-Aldrich; AB_477629	Anti-mouse, IgG + IgM HRP	1:5000	Dianova	116

### Statistical Analyses

Data of immunohistological and Western blot analyses were accomplished *via* Student’s *t*-test and presented as mean ± standard error mean (SEM) ± standard deviation (SD) using Statistica software (V13.3; StatSoft Europe, Hamburg, Germany). T-tests are presented as box whisker plots. Here, the mean is represented as a black square, SEM as a box, and SD as a whisker. All values represented in the text represent mean ± SD. RT-qPCR results were evaluated with the pairwise fixed reallocation and randomization test (REST software) and were presented as median ± quartile ± minimum/maximum (Pfaffl et al., 2002). Statistical significance is given by the *p*-value: *p* < 0.05 = ∗, *p* < 0.01 = ∗∗, and *p* < 0.001 = ∗∗∗. The exact number of experimental repetitions is given in the figure legends.

## Results

### Reduced Number of PNNs and Ectopic Shift of Aggrecan in Quadruple Knockout the Visual Cortex

**FIGURE 1:**
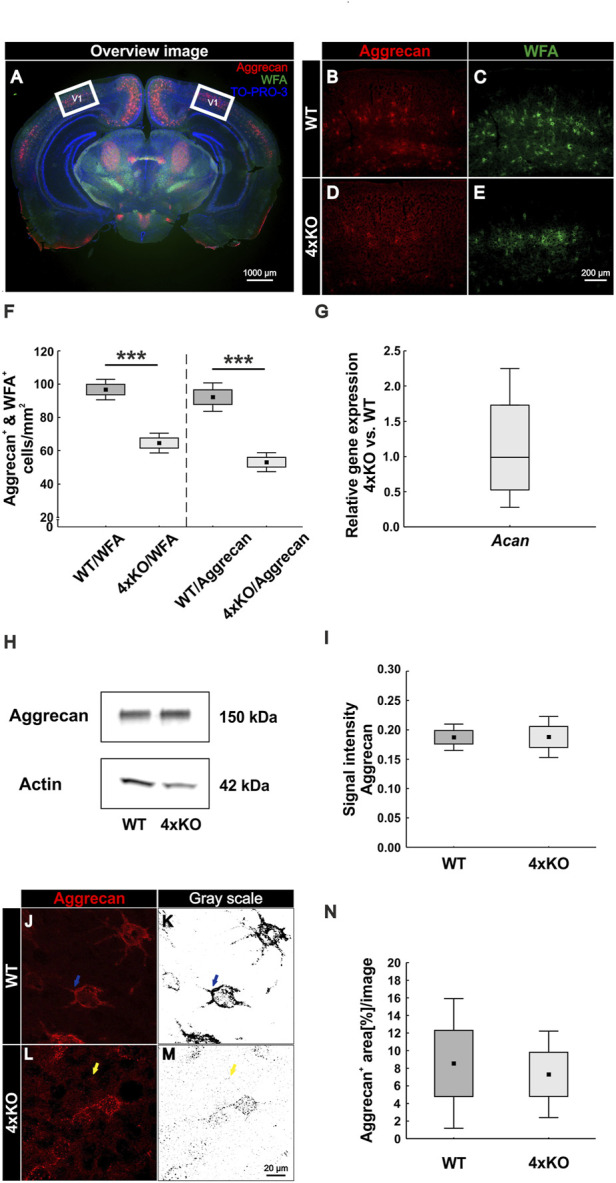
Diminished PNN organization in the visual cortex of quadruple knockout mice. **(A**–**E)** Immunohistochemical staining of PNNs in murine coronal brain sections with WFA (green) and anti-aggrecan (red). TO-PRO-3 was used as a nuclear marker. **(A)** Representative image demonstrating the selected area (white square) for cell counting and further analyses in the visual cortex. **(B**–**E)** Images of WFA-positive and aggrecan-positive PNN-enwrapped neurons were taken and counted. **(F)** A significantly reduced number of WFA-positive and aggrecan-positive cells (*p* < 0.001) in the V1 of quadruple knockout mice could be noticed (*N* = 7). **(G)** RT-qPCR analyses revealed a comparable *Acan* mRNA expression in the visual cortex of wildtype and quadruple knockout mice (*p* = 0.98, *N* = 6). **(H)** Western blot analysis of aggrecan protein levels in the V1. **(I)** No differences in the aggrecan protein band intensity were detectable in visual cortex tissue of wildtype and quadruple knockout mice (*p* = 0.98, *N* = 8). **(J** and **L)** For further analyses, aggrecan-positive interneurons were documented per laser-scanning microscopy with ×630 magnification. **(K** and **M)** Gray scale images of the analyzed aggrecan-positive signal. Blue arrows indicate PNN bound aggrecan in the wildtype, whereas yellow arrows indicate an ectopic shift from the perisynaptic space to the surrounding neuropil in the quadruple knockout mouse. **(N)** Quantification of the stained area showed no differences in the total aggrecan immunoreactivity between wildtype and knockout; 4xKO, quadruple knockout; V1, primary visual cortex; WFA, *Wisteria floribunda* agglutinin; WT, wildtype, ***p < 0.001 data are shown as mean ± SEM and SD, scale bar A = 1000 μm, scale bar B–E = 200 μm, and scale bar J–M = 20 µm.

Before analyzing PNNs in adult wildtype and quadruple knockout mice in the cerebral cortex, we examined the distribution of brevican, neurocan, tenascin-C, and tenascin-R in different cortical areas (Supplementary Figure S1). Neurocan appeared to generate the strongest staining pattern in the V1, whereas brevican was mostly detected in the RSC and only weakly in the V1. Tenascin-R presented the most positive cells in the RSC, but the signal intensity at individual positive cells was comparable between all regions. Tenascin-C is not expressed in the adult cortex and showed no specific immunoreactivity as expected. This study focused on conspicuities in the V1 of quadruple knockout mice, and results in other cortical areas are mainly presented in the supplements. Coimmunostaining with WFA and an antibody against aggrecan was performed on murine coronal brain sections of 16-week-old wildtype and quadruple knockout mice. WFA and aggrecan are well described as markers for the visualization of PNNs in the CNS (Hartig et al., 1992; Matthews et al., 2002). An area of interest was chosen (white square), where images were taken for further analyses of the V1 (Figure 1A). In this area, images with a higher magnification were captured, and WFA-positive and aggrecan-positive cells were counted (Figures 1B–E). Significantly reduced numbers of WFA-positive (wildtype: 96.75 ± 8.32, knockout: 64.81 ± 7.42; *p* < 0.001) and aggrecan-positive (wildtype: 92.21 ± 11.54 vs knockout: 53.14 ± 7.71; *p* < 0.001) PNN-enwrapped cells were counted in the quadruple knockout V1 (Figure 1F). Analyses of other cortical areas near the V1 also showed significant reductions in the number of PNN-enwrapped cells (Supplementary Figure S2). Interestingly, despite the reduced number of aggrecan-positive PNNs in the quadruple knockout, no differences in the aggrecan mRNA expression could be detected between the wildtype and quadruple knockout V1 (0.99-fold, *p* = 0.98, Figure 1G). Furthermore, aggrecan protein levels, detected as a prominent band at 150 kDa (Figure 1H) by Western blot analyses, were comparable between both genotypes (wildtype: 0.19 ± 0.03 a.u. vs knockout: 0.19 ± 0.05 a.u., *p* = 0.98, Figure 1I). Considering that the number of aggrecan-positive cells was reduced in the knockout, but aggrecan mRNA expression and protein levels did not differ from the wildtype, laser scanning microscopy with a higher magnification of immunostained aggrecan-positive PNNs was performed to compare the total aggrecan signal and localization (Figures 1J–M). The statistical evaluation of the aggrecan positive area showed no differences between the wildtype and knockout V1 (wildtype: 8.55 ± 9.2% aggrecan-positive area vs knockout: 7.31 ± 6.14% aggrecan-positive area, *p* = 0.79). It can be noted that the aggrecan signal in the gray scale image of the wildtype was strictly located in the perisynaptic area of the PNN-positive cells (Figure 1J, blue arrows). In contrast, the gray scale image of the knockout showed a weaker signal at the perisynaptic area and an aggrecan-positive signal in the neuropil (Figure 1M, yellow arrows). These data showed a reduced number of PNNs with no differences in the aggrecan mRNA and protein levels in the quadruple knockout but also indicate an ectopic shift of aggrecan from the perisynaptic space to the surrounding neuropil.

### Impaired PNN Structure in the V1 of Quadruple Knockout Mice

**FIGURE 2:**
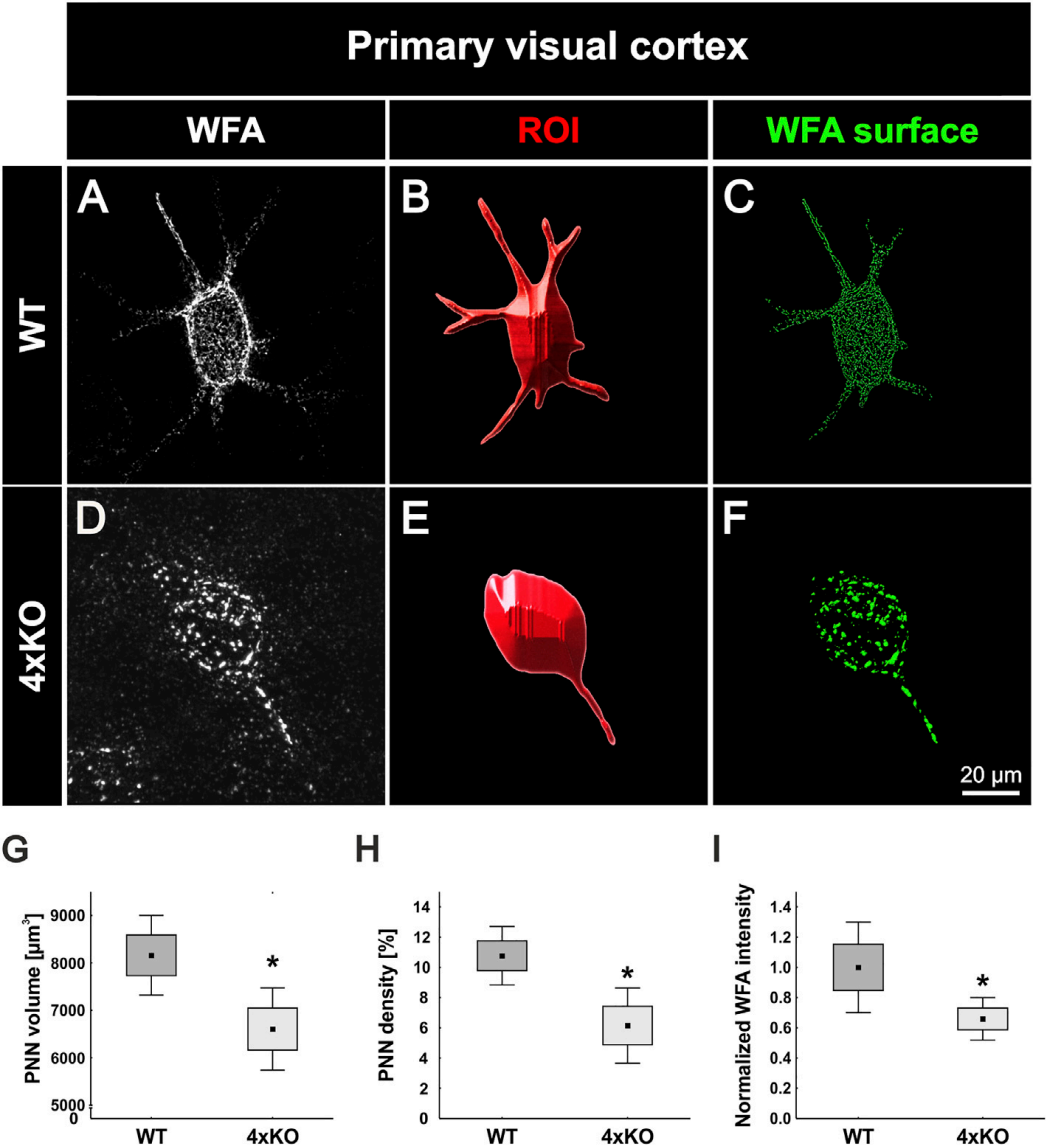
Structural characterization of PNNs in the V1 of wildtype and quadruple knockout mice using super-resolution SIM. **(A** and **D)** Representative SIM image of WFA-positive PNNs in the wildtype and quadruple knockout V1. As depicted exemplarily, the wildtype PNN showed a typical honeycomb structure with WFA-positive proximal dendrites. In contrast, the quadruple knockout PNN showed a disorganized structure with only one WFA-positive process. **(B** and **E)** Using IMARIS software, a 3D surface was generated manually containing the WFA-positive signal and the soma enveloped by the PNN, which was determined as ROI. **(C** and **F)** IMARIS surface technology was then used to create 3D surfaces of the WFA-positive signal inside the ROI best matching PNN anatomy. The WFA signal outside the ROI was suppressed. **(G)** Quantitative analysis revealed a significant reduction in the volume of quadruple knockout PNNs in comparison to wildtype PNNs (*p* < 0.05). **(H** and **I)**. In addition, PNN density (*p* < 0.05) and WFA intensity (*p* < 0.05) were significantly reduced in the quadruple knockout (*p* < 0.05), indicating fewer WFA-positive PNN components on the cell surface of PNN-enwrapped neurons in the quadruple knockout. 4xKO, quadruple knockout; ROI, region of interest; WFA, *Wisteria floribunda* agglutinin; WT, wildtype, *N* = 8, *p < 0.05 data are shown as mean ± SEM and SD, scale bar = 20 µm.

We showed that the number of PNNs in the quadruple knockout was significantly reduced, and aggrecan shifted to the surrounding neuropil. It is well described that brevican, neurocan, and Tnr are components of PNNs, and Tnc is associated with the PNN structure (Bekku et al., 2003; Galtrey et al., 2008; Morawski et al., 2014; Jakovljevic et al., 2021). Therefore, we investigated the impact of the quadruple knockout on the PNN structure in the V1 with high-resolution SIM. WFA was used as a marker because it showed a more distinct staining pattern than antibodies against aggrecan. PNNs in the wildtype visual cortex appeared with the typical honeycomb structure and accumulation on proximal neurites (Figure 2A). In contrast, quadruple knockout PNNs exhibited a disrupted structure and less intensely stained proximal neurites (Figure 2D). Images were then transferred to the IMARIS software for further analyses. A 3D surface of individual PNNs was generated and determined as ROI (Figures 2B and E). The WFA-positive signal outside the ROI was suppressed. Next, the volume of the ROI was measured *via* IMARIS, which reflects the volume of the PNN enwrapping an interneuron. We observed a significant reduction in the volume of the quadruple knockout PNNs (wildtype V1: 8161 ± 1213 μm^3^ vs knockout V1: 6606 ± 1254 μm^3^, *p* < 0.05, Figure 2G). In addition, the density of the PNNs was examined. Therefore, the “surface tool” of IMARIS was used again to obtain an accurate 3D structure of the WFA-positive signal inside of the ROI (Figures 2C and F). The sum of all volumes of WFA-positive signals at the 3D structure was then automatically measured, describing the volume of all WFA-positive PNN components surrounding the neuron. The volume of the WFA-positive PNN components was then divided by the volume of the overall PNN, enwrapping a neuron. Thus, we could identify the amount of WFA-positive PNN components in the overall volume and, therefore, the density of the PNNs. The wildtype revealed significantly denser PNNs in comparison to the quadruple knockout PNNs, reflecting a reduced distribution of PNN components surrounding the cell (wildtype V1: 10.77 ± 2.79% PNN density vs knockout V1: 6.15 ± 3.60%, *p* < 0.05, Figure 2H). In addition, the quadruple knockout PNNs showed a reduced WFA fluorescence intensity (wildtype: 1.00 ± 0.75% vs knockout: 0.66 ± 0.35%, *p* < 0.05, Figure 2I). In conclusion, the elimination of the four ECM genes not only led to a reduced number of PNNs but also the structure of the remaining knockout PNNs was severely disrupted.

### Disruption of PNNs Affects Synaptic Integrity

The important role of PNNs in synaptic homeostasis has been well described (Bukalo et al., 2001; Geissler et al., 2013; Bosiacki et al., 2019). PNNs can act as a physical barrier to prevent unspecific neuronal connections. Furthermore, PNN components can act as a binding partner for inhibitors of synaptic contacts, and they reduce the mobility of ionotropic receptors on the neuronal membrane (Saroja et al., 2014; Sala et al., 2015; van 't Spijker and Kwok, 2017). In this context, and given that the PNN structure in the quadruple knockout appeared disrupted, it was of interest to investigate synapse formation on PNN-enwrapped neurons in wildtype and quadruple knockout mice.

#### Reduced Number of Structural Inhibitory Synapses at Quadruple Knockout PNNs

**FIGURE 3:**
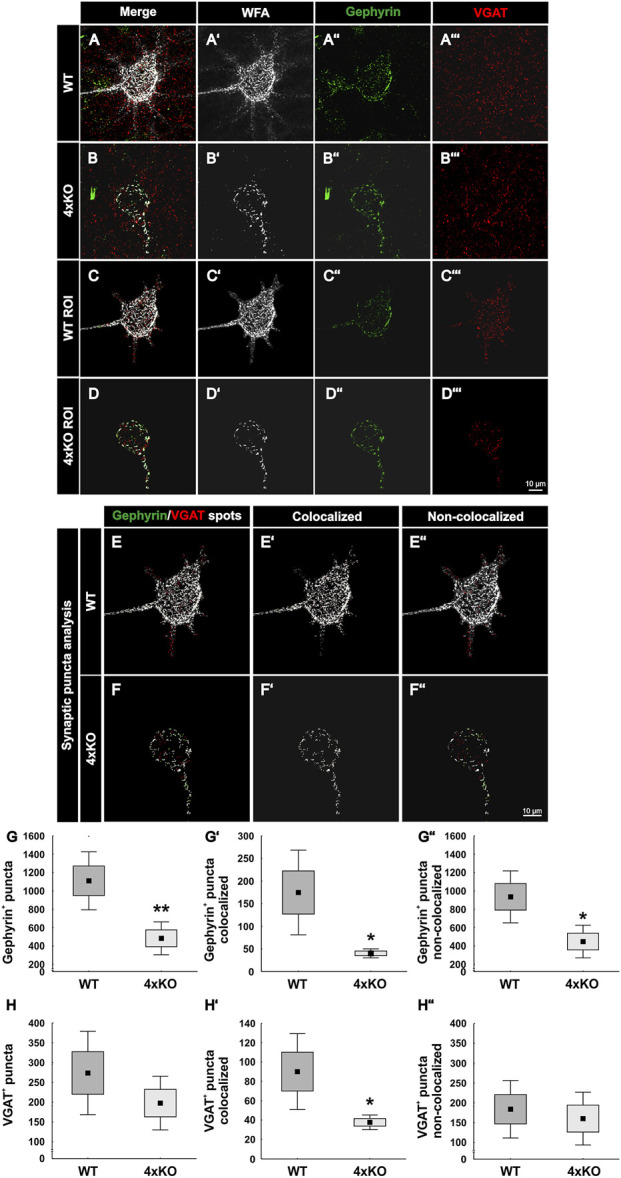
Distribution of inhibitory synaptic elements along PNNs in the V1 of wildtype and quadruple knockout mice. **(A** and **B’’’)** Immunohistochemical staining of wildtype and quadruple knockout PNNs and inhibitory synapses in the V1. WFA (white) was used as a specific marker for PNNs, antibodies against gephyrin (green) and VGAT (red) as specific markers for inhibitory postsynaptic elements or for inhibitory presynaptic elements, respectively. **(C** and **D’’’)** An ROI was generated, including solely a single PNN with its perforating inhibitory synapses. **(E** and **F’’)** Representative images of synaptic puncta represented as spots and checked for their localization *via* IMARIS. Colocalized gephyrin spots are represented in light green, VGAT spots in light red, and non-colocalized spots in dark green or rather dark red. **(G**–**G’’)** Statistical evaluation revealed a significant decrease in the total number of gephyrin-positive puncta (*p* < 0.05) and VGAT and gephyrin double-positive puncta (*p* < 0.05). In addition, the number of non-colocalized gephyrin-positive puncta was significantly reduced (*p* < 0.05). **(H**–**H’’)** Furthermore, the total amount of VGAT-positive puncta was comparable between wildtype and quadruple knockout (*p* = 0.25), but significantly fewer VGAT-positive and gephyrin-positive colocalized puncta were observed (*p* < 0.05). The number of non-colocalized VGAT-positive puncta showed no difference between wildtype and quadruple knockout (*p* = 0.65). 4xKO, quadruple knockout; ROI, region of interest; VGAT, vesicular GABA transporter; WT, wildtype, *N* = 8; **p < 0.01, data are shown as mean ± SEM and SD, scale bars = 10 μm.

For the analyses of inhibitory synapses on WFA-positive PNNs, specific antibodies against gephyrin and VGAT were used. Gephyrin and VGAT served as marker for inhibitory postsynaptic and presynaptic elements, respectively, in wildtype and quadruple knockout V1 (Figures 3A–B’’’). PNNs were singled out as described (Figure 2), and the synaptic contacts along the PNNs were analyzed (Figures 3C–D’’’). Signals localized outside the ROI were neglected. Synaptic puncta were examined corresponding to their localization. Therefore, spots were generated with the IMARIS software and the tool “Colocalize spots” was used to check the colocalization of pre- and postsynaptic markers (Figures 3E–F’’). Gephyrin-positive and VGAT-positive puncta which were located within a radius of 1 µm of each other were defined as colocalized (Supplementary Figure S3). We interpret pre- and postsynaptic puncta in such spatial proximity as part of a structural synapse (Pyka et al., 2011; Fogarty et al., 2013). Synaptic puncta without a counterpart within a radius of 1 µm were defined as non-colocalized. Most of the gephyrin-positive inhibitory postsynaptic spots seemed to be located at the soma of the cell, whereas inhibitory VGAT-positive presynaptic spots were evenly distributed along the soma and proximal dendrites in the wildtype and quadruple knockout. Immunostainings of gephyrin appeared to be particularly visible near the PNNs. High magnification images indicate the appearance of gephyrin-positive stainings right next to the WFA stained CSPGs of the PNN (Supplementary Figure S3). Gephyrin-positive spots are illustrated in green and VGAT-positive spots in red. If the spots were colocalized, they were illustrated in light green and light red, respectively (Figures 3E and F). Statistical analysis showed that the total number of gephyrin-positive puncta was significantly reduced at the quadruple knockout PNNs in comparison to the wildtype PNNs (wildtype PNNs: 1110.19 ± 456.26 gephyrin-positive puncta vs knockout PNNs: 483.36 ± 258.91 gephyrin-positive puncta, *p* < 0.01, Figure 3G). In contrast, the total number of VGAT-positive puncta along the PNNs did not differ between the wildtype and quadruple knockout (wildtype PNNs: 273.63 ± 151.51 vs. knockout PNNs: 197.68 ± 97.63, *p* = 0.25, Figure 3H). However, it is noticeable that the number of VGAT-positive inhibitory presynaptic puncta is clearly smaller than the number of gephyrin-positive inhibitory postsynaptic puncta. Statistical evaluation of gephyrin and VGAT colocalized spots showed a significant reduction in the number of colocalized inhibitory gephyrin-positive puncta in the quadruple knockout (wildtype PNNs: 174.80 ± 1135.01 gephyrin-positive puncta colocalized vs knockout PNNs: 40.25 ± 13.52 gephyrin-positive puncta colocalized, *p* < 0.05, Figure 3G’). Also, the number of colocalized inhibitory VGAT-positive puncta was significantly reduced at the quadruple knockout PNN in comparison to the wildtype PNN (wildtype PNNs: 90.04 ± 56.65 VGAT-positive puncta colocalized vs knockout PNNs: 37.60 ± 10.98 VGAT-positive puncta colocalized, *p* < 0.05, Figure 3H’). The reduced number of colocalized gephyrin and VGAT puncta, defined as part of a structural synapse, indicates a disturbed formation of inhibitory synapses in the quadruple knockout. In addition, the number of non-colocalized gephyrin-positive puncta was significantly reduced along quadruple knockout PNNs (wildtype PNNs: 935.39 ± 408.88 gephyrin-positive puncta non-colocalized vs knockout PNNs: 448.15 ± 257.11 gephyrin-positive puncta non-colocalized, *p* < 0.05, Figure 3G’’). In contrast, the number of non-colocalized VGAT-positive puncta was comparable between the wildtype and quadruple knockout (wildtype PNNs: 183.58 ± 104.55 VGAT-positive puncta non-colocalized vs. knockout PNNs: 160.08 ± 96.05 VGAT-positive puncta non-colocalized, *p* = 0.65, Figure 3H’’). The reduction of inhibitory synaptic puncta seems to affect, especially gephyrin-positive postsynaptic structures, whereas presynaptic VGAT-positive structures were only reduced regarding their co-localization with gephyrin, possibly as a consequence of the reduced number of gephyrin puncta. Analyses of the protein level and mRNA expression of both inhibitory synaptic marker in V1 tissue showed no differences between wildtype and quadruple knockout (Supplementary Figure S4). Therefore, the alteration in the distribution of inhibitory synaptic elements seemed to originate in a disturbed synaptic organization along quadruple PNNs and was not ascribed to an altered number of inhibitory synapses in quadruple knockout tissue. In sum, this result indicates a disturbed formation of structural inhibitory synapses at PNNs caused by the combined loss of brevican, neurocan, Tnc, and Tnr. In particular, the number of inhibitory postsynaptic elements and the formation of structural inhibitory synapses seemed affected.

#### Increase of Excitatory Synaptic Puncta on PNN-Enwrapped Quadruple Knockout Neurons

**FIGURE 4:**
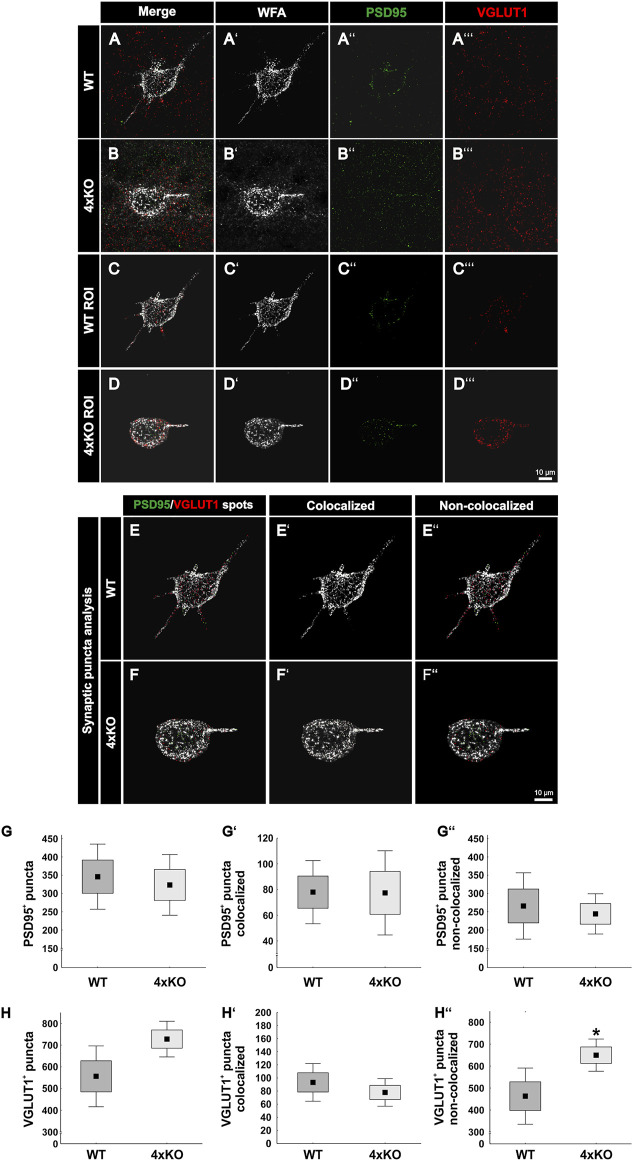
Expression of PSD95-positive and VGLUT1-positive synaptic puncta at wildtype and quadruple knockout PNNs in the V1. **(A** and **B’’’)** Representative SIM image representing immunohistochemical stainings of WFA-positive PNNs (white) and excitatory synapses with PSD95 as a marker for excitatory postsynapses (green) and VGLUT1 as a marker for excitatory presynapses (red). **(C** and **D**’’’**)** An ROI was generated, including single wildtype and quadruple knockout PNNs with their perforating excitatory synapses. **(E** and **F’’)** For quantitative evaluation, colocalized PSD95 spots are represented in light green, VGLUT1 spots in light red, and non-colocalized spots in dark green or rather dark red. **(G**–**G’’)** The total number of PSD95-positive synaptic puncta, the number of PSD95-positive and VGLUT1-positive colocalized synaptic puncta, and the number of non-colocalized PSD95-positive synaptic puncta along the PNNs were quantified. No difference in the total number of PSD95-positive synaptic puncta (*p* = 0.72) nor in the number of colocalized (*p* = 0.98) or non-colocalized PSD95-positive synaptic puncta (*p* = 0.69) between wildtype and quadruple knockout PNN was detected. **(H**–**H’’’)** In addition, the total number of excitatory presynaptic VGLUT1-positive synaptic puncta was comparable between quadruple knockout PNNs and wildtype PNNs (*p* <= 0.06). The number of colocalized VGLUT1-positive was similar in the wildtype and quadruple knockout (*p* = 0.41). However, non-colocalized VGLUT1-positive synapses were significantly reduced on quadruple knockout PNNs (*p* < 0.01). 4xKO, quadruple knockout; PSD95, postsynaptic density protein 95; ROI, region of interest; VGLUT1, vesicular glutamate transporter 1; WT, wildtype, *N* = 8; **p < 0.01, data are shown as mean ± SEM and SD, scale bars = 10 μm.

Next, WFA-positive PNNs in the V1 of wildtype and quadruple knockout mice were examined immunohistochemically for the distribution of excitatory synaptic elements. Therefore, antibodies against PSD95 as a specific marker for excitatory postsynaptic puncta and against VGLUT1 as a specific marker for excitatory presynaptic puncta have been used (Naito and Ueda, 1985; Hunt et al., 1996). To analyze excitatory synaptic elements along wildtype and knockout PNNs, a region of interest containing a single PNN was circumscribed (Figures 4A-D’’’). PSD95-positive and VGLUT1-positive signals outside the ROI were suppressed. Next, synaptic spots were generated to analyze the total number of excitatory synapses and evaluate their localization (Figures 4E–F’’). PSD95-positive and VGLUT1-positive puncta which were located within a radius of 1 µm were defined as colocalized. We interpret pre- and postsynaptic puncta in such spatial proximity as part of a structural synapse. The number of PSD95-positive excitatory postsynaptic puncta appeared unaltered in knockout mice (wildtype PNNs: 345.95 ± 128.25 PSD95-positive synaptic puncta vs knockout PNNs: 323.42 ± 119.68 PSD95-positive synaptic puncta, *p* = 0.72, Figure 4G). The localization of the PSD95-positive synaptic puncta was also similar. No significant differences were found in the number of colocalized puncta (wildtype PNNs: 78.12 ± 35.46 PSD95-positive puncta colocalized vs. knockout PNNs: 77.57 ± 47.27 PSD95-positive puncta colocalized, *p* = 0.98, Figure 4G’). Moreover, the number of non-colocalized PSD95-positive puncta was similar between wildtype and knockout PNN-enwrapped neurons (wildtype PNNs: 267.82 ± 132.31 PSD95-positive puncta non-colocalized vs knockout PNNs: 245.84 ± 80.25 PSD95-positive puncta non-colocalized, *p* = 0.69, Figure 4G’’). In contrast, VGLUT1-positive excitatory presynaptic puncta showed an altered distribution along the quadruple knockout PNNs as described as follows. The number of VGLUT1-positive puncta was comparable between PNN-enwrapped neurons of the quadruple knockout and the wildtype (wildtype PNNs: 556.43 ± 202.27 VGLUT1-positive puncta vs knockout PNNs: 727.90 ± 202.26 VGLUT1-positive puncta *p* = 0.06, Figure 4H). The number of colocalized VGLUT1-positive synaptic puncta along the PNNs seemed unaltered (wildtype PNNs: 93.29 ± 41.52 colocalized VGLUT1-positive puncta vs knockout PNNs: 77.94 ± 30.36 colocalized VGLUT1 positive puncta, *p* = 0.41, Figure 4H’). Furthermore, a strong increase in the number of non-colocalized VGLUT1-positive puncta could be observed in association with quadruple knockout PNNs compared to wildtype PNNs (wildtype: 463.13 ± 184.49 VGLUT1-positive puncta non-colocalized vs knockout PNNs: 649.96 ± 105.77 VGLUT1-positive puncta non-colocalized, *p* < 0.05, Figure 4H’’). Similar to the inhibitory synaptic markers, analyses of the protein and mRNA expression level of excitatory markers in the V1 tissue were comparable between wildtype and quadruple knockout (Supplementary Figure S5); thus, supporting the impression that the altered synaptic distribution along the PNNs was the consequence of an impaired synaptic organization and not the consequence of an altered number of synapses. In conclusion, these observations led to the assumption that the deletion of the four quadruple PNN constituents resulted in an increase of excitatory synaptic elements along the PNNs. However, this increase appeared restricted to delocalized excitatory presynaptic puncta, whereas excitatory postsynaptic puncta and colocalized excitatory presynaptic puncta appeared unchanged, indicating a regular distribution of structural excitatory synapses on quadruple knockout PNNs.

### Loss of Parvalbumin-Positive Interneurons in Quadruple Knockout Mice

**FIGURE 5:**
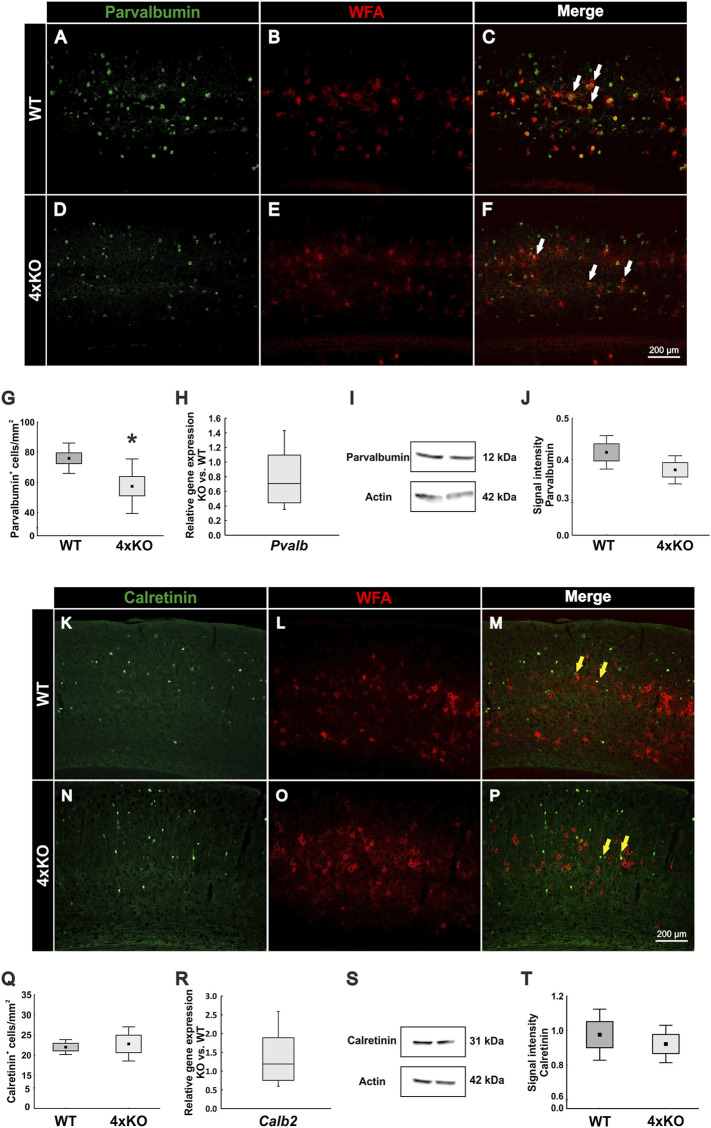
Analyses of parvalbumin-positive and calretinin-positive interneuron populations in the V1 of wildtype and quadruple knockout V1. **(A**–**F)** Representative coronal V1 brain sections of wildtype and quadruple KO double-labeled using a specific antibody against parvalbumin and WFA in the V1. Parvalbumin-positive fast-spiking interneurons were mainly PNN-enveloped (white arrows). **(G)** The number of parvalbumin-positive cells was significantly reduced in the quadruple knockout (*p* < 0.05, *N* = 8). **(H)** RT-qPCR analyses revealed a comparable *Pvalb* mRNA expression in the visual cortex of wildtype and quadruple knockout mice (*p* = 0.09, *N* = 6). **(I)** Western blot analyses of parvalbumin in the visual cortex of wildtype and quadruple knockout. A prominent band was detected at 12 kDa. **(J)** Statistical analyses revealed comparable parvalbumin protein levels between wildtype and quadruple knockout (*p* = 0.14, *N* = 7). **(K**–**P)** Representative coronal V1 brain sections of wildtype and quadruple knockout mice double-labeled with a specific antibody against calretinin and WFA. In contrast to parvalbumin-positive interneurons, calretinin-positive fast-spiking interneurons were not enveloped by PNNs (yellow arrows). **(Q)** Statistical analyses showed a comparable number of calretinin-positive interneurons in the wildtype and quadruple knockout V1 (*p* = 0.75, *N* = 8). **(R)** *Calb2* mRNA expression levels were comparable between wildtype and quadruple knockout (*p* = 0.38, *N* = 6). **(S)** Western blot analyses of calretinin in V1 tissue of wildtype and quadruple knockout. **(T)** No differences in the calretinin protein band intensity were detectable in visual cortex tissue of wildtype and quadruple knockout mice (*p* = 0.57, *N* = 8). 4xKO, quadruple knockout; *Calb2*, calbindin 2 (calretinin); *Pvalb*, parvalbumin; WT, wildtype; WFA, *Wisteria floribunda* agglutinin, *p < 0.05, data are shown as mean ± SEM and SD, scale bars = 200 μm.

As a next step, it appeared of interest to analyze if the disruption of the PNNs and the synaptic modification along the quadruple knockout PNNs affects the cell population enwrapped in PNNs. In the cortex, PNNs mainly enwrap fast-spiking parvalbumin-positive interneurons (Hartig et al., 1992; Dityatev et al., 2007). To visualize these interneurons, we immunohistochemically double-stained WFA-positive PNNs with antibodies against parvalbumin in wildtype and quadruple knockout V1 (Figures 5A–F). Most of the parvalbumin-positive interneurons are covered by PNNs, as indicated by the white arrows. The statistical evaluation showed a significant decrease in the number of parvalbumin-positive cells in the V1 of quadruple knockout mice in comparison to the wildtype (wildtype: 75.94 ± 10.07 parvalbumin-positive cells/mm^2^ vs knockout: 57.37 ± 18.07, *p* < 0.05, Figure 5G). mRNA levels of parvalbumin in the V1 of quadruple knockout mice were comparable to the wildtype (0.71-fold, *p* = 0.09, *N* = 6, Figure 5H). In addition, parvalbumin protein levels detected as a prominent band at 12 kDa were comparable in the V1 of wildtype and quadruple knockout mice (wildtype: 0.41 ± 0.05 a.u. vs. knockout: 0.38 ± 0.04 a.u., *p* = 0.14, *N* = 7, Figures 5I and J). Of particular interest is that this reduction in the number of parvalbumin-positive interneurons was limited to the V1 area. Analyses of other cortex areas adjacent to the V1 showed no difference in the number of parvalbumin-positive cells between wildtype and knockout (Supplementary Figure S6). Given such a reduction of parvalbumin-positive interneurons, we also examined other interneuron populations in the V1 of wildtype and quadruple knockout. Another marker used for classifying interneurons in the visual cortex is the calcium-binding protein calretinin (Barinka and Druga, 2010). But in contrast to parvalbumin-positive interneurons, calretinin-positive interneurons are not enwrapped by PNNs. To analyze the number of calretinin-positive interneurons immunohistochemical labeling with specific antibodies against calretinin was performed (Figures 5K–P). In contrast to parvalbumin-positive interneurons, calretinin-positive interneurons appeared as not enwrapped by PNNs and as most in close proximity to PNN-enwrapped neurons (yellow arrows). In the case of the calretinin-positive interneuron population, no difference in the number of cells was detected in the V1 of both genotypes (wildtype: 22.00 ± 2.48 calretinin-positive cells/mm^2^ vs knockout: 22.79 ± 5.71 calretinin-positive cells/mm^2^, *p* = 0.75, Figure 5Q). In addition, in other cerebral cortex areas, calretinin-positive interneuron populations did not differ between wildtype and quadruple knockout (Supplementary Figure S7). *Calretinin* mRNA expression in the V1 of wildtype and quadruple knockout mice was also comparable (1.2-fold, *p* = 0.38, Figure 5R). Furthermore, similar calretinin protein levels, detected as a prominent band at 31 kDa (Figure 5S) by Western blot analyses, were found in wildtype and quadruple knockout V1 tissue (wildtype: 0.976 ± 0.21 a.u. vs knockout: 0.921 ± 0.16 a.u., *p* = 0.57, Figures 5S and T). In summary, these results showed that the parvalbumin-positive interneuron population was significantly reduced in the V1 of quadruple knockout mice, whereas the calretinin-positive interneuron population was comparable to the wildtype population. Whether this apparent loss of parvalbumin-positive interneurons is connected to the disrupted structure of the associated PNNs needs further investigation. It is worth noting that the reduced number of parvalbumin-positive cells was restricted to the V1, whereas other cortical areas seemed unaffected.

### Lack of Otx2 in the V1 as Possible Trigger for Reduced Parvalbumin Expression

**FIGURE 6:**
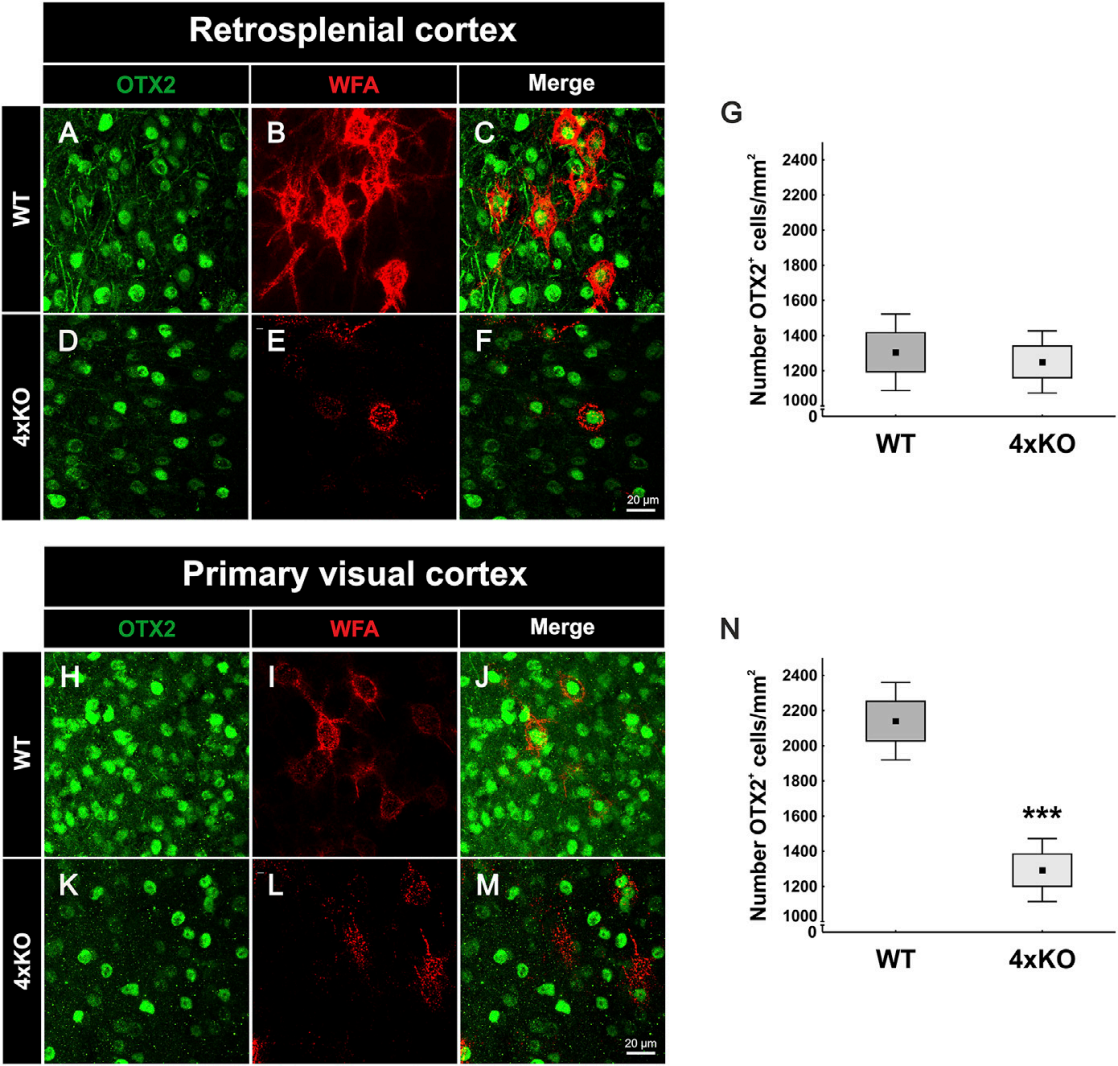
Expression of Otx2 in the cerebral cortex of wildtype and quadruple knockout mice. **(A-F, H-M)** Representative images of Otx2-positive cells (green) and WFA-positive PNNs (red) in the RSC and V1 of wildtype and quadruple knockout mice. PNN-enwrapped neurons and also other cells showed Otx2 accumulation at the soma. **(G)** Counting of Otx2-positive cells in the RSC revealed a comparable number in wildtype and quadruple knockout tissue (*p* = 0.7, *N* = 8). **(N)** In contrast, statistical evaluation of Otx2-positive cells in the V1 showed a strong reduction in the number of Otx2-positive cells of quadruple knockout mice in comparison to the wildtype (*p* < 0.001, *N* = 8). 4xKO, quadruple knockout; Otx2, orthodenticle homeobox 2; RSC, retrosplenial cortex; V1, primary visual cortex; WFA, *Wisteria floribunda* agglutinin; WT, wildtype, ***p < 0.001 data are shown as mean ± SEM and SD, scale bars A = 20 µm.

The remarkable reduction of the parvalbumin-positive interneuron population in the quadruple knockout V1 raised the question of potential causes. In this context, it is worth considering the possibility that the selective elimination of parvalbumin-positive interneurons might be causally linked to the structural modification of the associated PNNs in the quadruple knockout mutant. Beyond this option, other factors influencing parvalbumin-positive interneuron maturation might be considered. Otx2 is a transcription factor involved in parvalbumin-positive cell maturation. The protein is not synthesized by parvalbumin-positive cells in a cell-autonomous fashion but rather transferred from other CNS areas, binds with high affinity to PNNs and subsequently accumulates in the interneurons (Beurdeley et al., 2012; Sugiyama et al., 2008). The emergence of Otx2 correlates with parvalbumin-positive cell development across cortical regions. Otx2 localization in parvalbumin-positive cells in auditory and medial prefrontal cortexes is required for the onset and closure of critical period plasticity (Lee H. H. C. et al., 2017; Sakai et al., 2017). Therefore, Otx2 is a key regulator of parvalbumin-positive cell maturation across the cortex. Because of the reported role of PNNs in the trans-neuronal transfer of Otx2, we hypothesized that the structural deficits in the quadruple knockout PNNs should translate into deficits in Otx2 uptake. To test this hypothesis, immunohistochemical double-staining of Otx2 and WFA was performed in coronal brain sections of wildtype and quadruple knockout mice and captured *via* laser-scanning microscopy (Figures 6A–F, H-M). To determine a possible influence of Otx2 on parvalbumin-positive interneuron populations, two different cortical areas were examined. On the one hand, the V1, where a loss of parvalbumin-positive cells in quadruple knockout mice was observed, and on the other hand, the RSC, where the number of parvalbumin-positive cells was comparable between wildtype and quadruple knockout (Figure 5 and Supplementary Figure S6). The statistical analyses of the Otx2-positive cell number in these areas revealed significant differences. Comparable numbers were seen in the wildtype RSC compared to the quadruple knockout RSC (wildtype RSC: 1305.00 ± 314.37 Otx2-positive cells/mm^2^ vs knockout RSC: 1250.00 ± 255.33 Otx2-positive cells/mm^2^, *p* = 0.71, Figure 6G. In contrast, a significant reduction in the number of Otx2-positive cells was counted in the V1 of quadruple knockout mice compared to the wildtype V1 (wildtype V1: 2140.00 ± 318.89 Otx2-positive cells/mm^2^ vs knockout V1: 1292.50 ± 258.56 Otx2-positive cells/mm^2^, *p* < 0.001, Figure 6N). In conclusion, these data showed that the loss of Otx2 was restricted to the V1 in quadruple knockout mice, whereas the RSC seemed unaffected, paralleling the results regarding the parvalbumin-positive cell populations. In the light of these findings, the striking diminution of Otx2 in the V1 of quadruple knockout might represent a plausible cause for the loss of parvalbumin-positive interneurons. It should be noted that both cortical areas showed a strong disruption of PNNs (Figure 1 and Supplementary Figure S2). To analyze PNN mediated internalization of Otx2, we measured the intensity means of Otx2 and WFA in double-positive cells in the RSC and V1 of wildtype and quadruple knockout mice (Supplementary Figure S8). WFA intensities in the V1 were already measured with IMARIS (Figure 2), but to preserve comparability between the results of RSC and Otx2 estimates were repeated with the “CellProfiler” software. WFA intensity was significantly reduced in the RSC and V1 of quadruple knockout mice in comparison to the wildtype. These results agree with the observed impairment of PNN structure in the quadruple knockout. Surprisingly, the measured Otx2 intensity in RSC and V1 was comparable between quadruple knockout and wildtype animals. This indicates a similar Otx2 internalization despite the disturbed PNN structure. This rules out the idea that PNN disruption *per se* is the cause for parvalbumin- and Otx2-positive cell loss in our model. Instead, the reduced capacity of modified PNNs to serve as vehicles for Otx2 transfer presumably compromised the vision-related axonal network where Otx2 plays an important signaling role (Bernard and Prochiantz, 2016).

## Discussion

### Elimination of Brevican, Neurocan, Tenascin-C, and Tenascin-R Leads to a Reduced Number of PNNs in the Cerebral Cortex

In this study, we observed a diminished number of PNN-enwrapped cells in the V1 of quadruple knockout mice (Figure 1F). In addition, other cerebral cortical areas close to the V1 showed a reduction in the amount of PNN wearing neurons (Supplementary Figure S2). More detailed analyses of the quadruple knockout PNN structure *via* SIM provided evidence for a strong disruption in the composition of these PNNs (Figures 2A and D). Single knockout mice lacking either brevican, neurocan, Tnc or Tnr have been described to exhibit altered PNN structures (Faissner et al., 2010). The distribution of carbohydrate epitopes of PNNs on Tnr knockout hippocampal interneurons is abnormal and also a disruption of PNN structure in the cerebral cortex was shown, indicating a disturbance of the molecular scaffolding of ECM components in Tnr knockout PNNs (Weber et al., 1999; Bruckner et al., 2000). Brevican knockout PNNs appeared to be less pronounced and less concentrated near the plasma membrane (Brakebusch et al., 2002). Neurocan knockout mice showed a distinct PNN staining pattern in the olfactory bulb compared to their wildtype littermates, but in the cortex, a comparable general appearance and frequency of this net-like structure between wildtype and neurocan knockout mice were observed (Zhou et al., 2001; Hunyadi et al., 2020). In fact, Tnc knockout mice have been described as having reduced staining patterns of PNNs compared to wildtype animals, although Tnc is not a direct PNN component and only occurs in close proximity to PNNs (Stamenkovic et al., 2017; Jakovljevic et al., 2021). Compared to the quadruple knockout model, the disturbance of PNN structure in single knockouts appeared to be relatively mild. To our knowledge, no reduction in the number of PNNs is described respectively investigated in the single knockout cortices. This leads to the assumption, that not a single molecule but rather the perturbed interactions between several ECM constituents in the quadruple knockouts cause the intense impairments in structure and number of PNNs. We cannot clarify in this study if the reduced number of PNNs came about by a failure of PNN condensation during development or as a consequence of later disorganization of PNNs. An indication for a reduced number of PNNs might be reflected by the observed ectopic shift of aggrecan in the quadruple knockout. Even though the number of aggrecan-positive PNNs was strongly reduced, mRNA expression, protein level and also immunoreactivity for aggrecan in regard to the overall V1 tissue were similar to WT V1 tissue. This indicates no loss of aggrecan but rather an ectopic shift from the perisynaptic space to the surrounding neuropil of aggrecan in the quadruple knockout V1. It has been shown in mice that the knockout of aggrecan leads to an elimination of PNNs in an *in vitro* cortical culture model and in organotypic slice cultures and also to an ablated PNN structure *in vivo* (Giamanco et al., 2010; Rowlands et al., 2018). In addition, previous studies showed that the N-acetylgalactosamine-binding *Wisteria floribunda agglutinin* can detect components related to chondroitin sulphate proteoglycans (Bruckner et al., 2003). Brevican immunoreactivity can be found in the neuropil and PNNs in cortical layers. In the retrosplenial cortex, a subpopulation of brevican and WFA double-positive PNNs is present. But also, WFA-positive PNNs without brevican labeling were detected. Therefore, brevican-positive PNNs can be detected by WFA, but not every WFA-positive PNN represents a brevican positive PNN (Ajmo et al., 2008). So, beyond the ectopic shift of aggrecan, also absence of brevican and neurocan in the quadruple knockout and accompanying missing binding partners for WFA might disrupt PNN structure and number. Nevertheless, these observations stage aggrecan as the main functional constituent and orchestrator of PNN assembly.

### The Structure of Cortical PNNs Is Strongly Disturbed in the Quadruple Knockout

Trimeric Tnr can bind three lecticans simultaneously and has been shown to stabilize PNN formation by clustering aggrecan (Galtrey et al., 2008; Morawski et al., 2014). Furthermore, trimeric Tnr and hexameric Tnc can cross-link the G3 domains of lecticans and thus tie up the extracellular network (Lundell et al., 2004). Considering the loss of two important scaffolding proteins of the tenascin family, it seems comprehensible that quadruple knockout mice are unable to form a proper PNN structure, which is reflected by the reduced volume, density and WFA intensity of PNNs. It is conceivable that a tampered aggrecan accumulation on PNNs leads to a complete failure of ECM condensation. However, due to the extensive molecular heterogeneity of residual PNNs, aggrecan might accumulate through HAPLN binding and contribute to PNNs with disturbed structure, resulting in a reduced number but not a total loss of PNNs in the quadruple knockout. These results are consistent with previous *in vitro* studies of hippocampal cultures of quadruple knockout mice, which also revealed a disturbed PNN organization. However, the number of PNN-enwrapped neurons has not been investigated in these cases (Geissler et al., 2013). Another interesting observation in the PNN structure of quadruple knockout mice was the reduced number of PNN-enwrapped neuronal extensions (Supplementary Figure S2). Quadruple knockout PNNs tended to surround only one extension, while wildtype PNNs had three to four extensions enwrapped. Since the quadruple knockout had only one PNN-enwrapped extension, it might be the axon, which is the only neuronal extension that has a unique function and appearance compared to the dendrites. In this case, this would indicate a different PNN composition around the axon in comparison to the dendrites, with an important role for the four ECM molecules in the dendritic extracellular environment but not the axonal ECM. However, previous studies have shown a similar composition of the perineuronal micro milieu for diverse cellular domains, contradicting this idea (Bruckner et al., 2006).

### PNNs in the V1 of Quadruple Knockout Mice Exhibit an Altered Synaptic Distribution Pattern

As previously mentioned, PNNs are an important organizer of synaptic integrity along inhibitory neurons in the cortex (Sigal et al., 2019). *In vivo* studies in the dorsal dentate gyrus of quadruple knockout mice have already shown an altered synaptic function and plasticity (Jansen et al., 2017). Also, hippocampal PNN-enwrapped neurons had a modified number of excitatory and inhibitory synapses, as shown by *in vitro* experiments (Gottschling et al., 2019). In our study, we could show that the number of inhibitory and excitatory synaptic elements and their organization is rather different along quadruple knockout PNNs in comparison to wildtype PNNs in the V1. Inhibitory synapses appeared to be more strongly affected than excitatory synapses. In particular, gephyrin-positive inhibitory synaptic puncta were reduced in their number on quadruple knockout PNN-enwrapped neurons. In addition, colocalized gephyrin-positive and VGAT-positive puncta were significantly reduced. Taking into account, that the overall number of VGAT-positive puncta along PNNs was comparable between wildtype and quadruple knockout, failed organization of inhibitory synaptic elements in the quadruple knockout might be a consequence of a reduction of inhibitory postsynaptic structures and therefore missing interaction partners for inhibitory presynaptic structures. Apparently, the disruption of the PNNs interferes with the organization of inhibitory synapses. In contrast, PNN disruption in the quadruple knockout V1 did not affect the organization of structural excitatory synapses. The number of structural PSD95-positive and VGLUT1-positive synaptic elements was comparable to wildtype PNNs. However, the number of VGLUT1-positive delocalized synaptic puncta along the quadruple PNN happened to be strongly increased. PNNs are described to restrict synaptic plasticity by inhibiting axonal sprouting or by acting as a passive diffusion barrier for cell surface molecules like neurotransmitter receptors (Pizzorusso et al., 2002; Frischknecht and Gundelfinger, 2012; Carstens et al., 2016) Therefore, an increased occurrence of VGLUT1 puncta in the quadruple knockout PNN might be a consequence of their severe impairments in structure. However, the number of PSD95-positive puncta was comparable to wildtype PNNs accompanied by a comparable colocalization of PSD95 and VGLUT1. This suggests that an increased number of excitatory presynaptic elements arrive at quadruple knockout PNNs, but because of a limited amount of excitatory postsynaptic elements at the PNN, most of them remain delocalized. In conclusion, the synaptic distribution at quadruple knockout PNNs showed an imbalance of excitatory and inhibitory synaptic organization. Similar results were found in *in vitro* studies of hippocampal PNN-enwrapped neurons isolated from quadruple knockout mice. Here, a significantly reduced number of inhibitory and an increased number of excitatory synaptic molecules along the PNNs were described (Gottschling et al., 2019). In addition, *in vivo* studies showed that the depletion of PNNs in mature neuronal networks decreases the density of inhibitory synapses (Dzyubenko et al., 2021). mRNA and protein levels of the analyzed synaptic markers in the whole V1 tissue samples, on contrary, showed generally no differences between wildtype and quadruple knockout. This suggests that the altered synaptic organization at the PNNs results from the disruption of its structure and not an effect concerning all the cells in the V1. The organization of synaptic connections by PNNs is well described (Fawcett et al., 2019). They can act as a physical barrier to prevent axons of other neurons to connect with the body of its enwrapped neuron (Corvetti and Rossi, 2005; Pyka et al., 2011). An impaired balance between excitation and inhibition contributes to the pathophysiology of autism and related neuropsychiatric disorders (Nelson and Valakh, 2015; Lee E. et al., 2017; Sohal and Rubenstein, 2019). Furthermore, interactions between PNNs and parvalbumin interneurons are altered in mouse models of autism (Xia et al., 2021). In this context, the quadruple knockout mouse model could be interesting for studies of neurological disorders in the future.

### The Quadruple Knockout Leads to a Reduced Number of Parvalbumin-Positive, but Not Calretinin-Positive Inhibitory Neurons in the V1

The PNN disruption and synaptic alterations in quadruple knockout V1 were accompanied by a reduction in the parvalbumin interneurons. In the visual cortex, PNNs enwrap mainly parvalbumin-positive interneurons (Hartig et al., 1992; Aronitz et al., 2021). In contrast, calretinin interneurons, which appeared not to be ensheathed by PNN, displayed no alteration in their number of cells in comparison to the wildtype V1. A protective role of PNNs for their parvalbumin neurons is described. In this regard, PNNs can act as a protective shield for parvalbumin interneurons against oxidative stress and potential neurochemical stimuli (Suttkus et al., 2012; Cabungcal et al., 2013; Morawski et al., 2014; Reichelt et al., 2019). Because of their fast-spiking properties, parvalbumin interneurons have a high metabolic demand, which renders them sensitive to oxidative stress. Other interneurons, such as calretinin-positive interneurons, are not particularly sensitive to oxidative stress (Cabungcal et al., 2013). Missing protection by PNNs for parvalbumin interneurons leading to their loss in the quadruple knockout V1 seems plausible, considering the disturbed structure and reduced number of PNNs. In addition, although the occurrence of oxidative stress in this animal model is not documented, glutamate is considered responsible for most oxidative stress induction in the mammalian brain (Herrera et al., 2001). This scenario is consistent with the increased number of VGLUT1-positive synaptic puncta at quadruple knockout PNNs. In contradiction with this interpretation, other cortical areas, where the number of PNNs was reduced as well, did not display a reduction in the number of parvalbumin interneurons.

### Reduced Number of Otx2-Positive Cells in the V1 of Quadruple Knockout Mice

Another important factor for parvalbumin maturation is Otx2 (Lee H. H. C. et al., 2017; Apulei et al., 2019). Otx2 is persistently internalized by parvalbumin-positive interneurons through PNN binding. In addition, the disruption of Otx2 localization to parvalbumin interneurons reduces parvalbumin and PNN expression (Beurdeley et al., 2012). Interestingly, similar to parvalbumin interneurons, the number of Otx2-positive cells was reduced in the V1 but not in the RSC of quadruple knockout mice. It appears likely that the reduced number of Otx2-positive cells is causally linked to the missing parvalbumin interneurons in the quadruple knockout V1. However, the reduction of Otx2-positive cells appeared considerably more extensive than the reduction in parvalbumin interneurons. It is postulated that the transfer of Otx2 from visual pathways to the V1 depends on visual experience. Enucleation of the eyes or dark-rearing from birth reduces Otx2 protein levels, which leads to a loss of Otx2-positive cells, weakens parvalbumin expression, and results in a reduced number of WFA-labeled cells compared to intact animals. In addition, calretinin-positive cells were not affected in their number (Sugiyama et al., 2008). Thus, the interplay between PNNs, interneurons and Otx2 in the V1 of quadruple knockout mice seems reminiscent of mouse models with deprivation in visual experience and input. PNN degradation by digesting CSPGs coupled to visual sensory input altered synapse selectively onto parvalbumin interneurons and showed PNN control of visual processing (Faini et al., 2018). Nevertheless, a disturbed PNN structure was observed not only at the V1 of quadruple knockout mice but also in the other examined cortical areas. In addition, despite their impaired structure, PNN mediated Otx2 internalization appeared undisturbed. A possible explanation is that quadruple knockout mice did not entail a total loss of PNNs, but some rudimental aggrecan-positive PNNs were still formed. The internalization of Otx2 into the neurons might be mediated by the remaining PNN bound aggrecan proteins. Previous studies showed that differential glycosylation of aggrecans controls the formation of microdomains in single PNNs and their specific binding of Otx2 (Nadanaka et al., 2020). So, given the feedback loop between PNNs and Otx2, remaining PNNs in the quadruple knockout could have aggrecan-cores with Otx2 binding sites, which allows them to mediate the internalization of Otx2 into their enwrapped neuron. Internalized Otx2, in turn, promotes the preservation of PNNs. In contrast, quadruple knockout PNNs with differently glycosylated aggrecan cores, could miss Otx2 binding sites and therefore fail to preserve the PNNs. This could also explain why a small population of PNNs is present in the quadruple cortex but in comparison to the wildtype, the number is reduced strongly. This still raises the question of why the number of Otx2-positive cells in the V1 of quadruple knockout mice is reduced but not in the RSC. This might have its origin in the way of Otx2 transport into cortical structures. Otx2 reaches the visual cortex as follows: *In vivo*, Otx2 is synthesized in retinal bipolar cells. From bipolar cells, it is transferred to retinal ganglion cells which are devoid of Otx2 mRNA. Transiting *via* neurons in the lateral geniculate nucleus it is transported to the visual cortex. Thereby, Otx2 travels *via* axonal transport mechanisms along the visual pathway (Edelstein and Smythies, 2013). This transport is experience-dependent. In addition, Otx2 is released from the choroid plexus epithelium into the cerebrospinal fluid and transferred into distant cerebral cortex areas (Spatazza et al., 2013; Planques et al., 2019; Planques et al., 2021). In summary, the transfer of Otx2 along visual structures seems disturbed. This might result from impaired visual processing or from the absence of tenascin-C and tenascin-R and the consequence for axon guidance. Removal of these molecules in the quadruple knockout could disrupt axonal transport mechanisms of Otx2 along visual pathways. In contrast, Otx2 from choroid plexus origin seems to reach other cortical areas in a similar manner as in the wildtype. Therefore, brevican, neurocan, Tnc, and Tnr seem to be important regulators of visual system integrity, especially by virtue of their role as PNN components.

Regarding this data, future studies on the impact of the knockout of the four ECM molecules on visual processing might be interesting for a better understanding of ECM involvement in the organization of visual pathways.

## Data Availability

The raw data supporting the conclusions of this article will be made available by the authors, without undue reservation.

## Abbreviations

AMPA, α-amino-3-hydroxy-5-methyl-4-isoxazolepropionic acid; bp, base pairs; BSA, bovine serum albumin; CNS, central nervous system; CSPG, chondroitin sulfate proteoglycan; ECM, extracellular matrix; for, forward; GABA, gamma-aminobutyric acid; HAPLN, hyaluronan and proteoglycan link protein; h, hour(s); KO, knockout; mEPSCs, miniature excitatory postsynaptic currents; mIPSCs, miniature inhibitory postsynaptic currents; min, minute; Otx2, orthodenticle homeobox 2; PBS, phosphate-buffered saline; PFA, paraformaldehyde; PNN, perineuronal net; PSD95, postsynaptic density protein 95; rev, reverse; ROI, region of interest; RRID, Research Resource Identifier; RSC, retrosplenial cortex; RT-qPCR, quantitative real-time PCR; SD, standard deviation; SDS, sodium dodecyl sulfate; SEM, standard error mean; SIM, structured illumination microscopy; TBS, tris-buffered saline; Tnc, tenascin-C; Tnr, tenascin-R; V1, primary visual cortex; VGAT, vesicular GABA transporter; VGLUT1, vesicular glutamate transporter 1; WFA, *Wisteria floribunda* agglutinin; WT, wildtype.

## References

[B1] Ajmo J. M. Eakin A. K. Hamel M. G. Gottschall P. E. (2008). Discordant Localization of WFA Reactivity and brevican/ADAMTS-Derived Fragment in Rodent Brain. BMC Neurosci. 9, 14. 10.1186/1471-2202-9-14 18221525 PMC2263047

[B2] Apulei J. Kim N. Testa D. Ribot J. Morizet D. Bernard C. (2019). Non-cell Autonomous OTX2 Homeoprotein Regulates Visual Cortex Plasticity through Gadd45b/g. Cereb. Cortex 29, 2384–2395. 10.1093/cercor/bhy108 29771284

[B3] Aronitz E. M. Kamermans B. A. Duffy K. R. (2021). Development of Parvalbumin Neurons and Perineuronal Nets in the Visual Cortex of normal and Dark‐exposed Cats. J. Comp. Neurol. 529, 2827–2841. 10.1002/cne.25127 33576496

[B4] Barinka F. Druga R. (2010). Calretinin Expression in the Mammalian Neocortex: a Review. Physiol. Res. 59, 665–677. 10.33549/physiolres.931930 20406030

[B5] Bekku Y. Saito M. Moser M. Fuchigami M. Maehara A. Nakayama M. (2012). Bral2 Is Indispensable for the Proper Localization of Brevican and the Structural Integrity of the Perineuronal Net in the Brainstem and Cerebellum. J. Comp. Neurol. 520, 1721–1736. 10.1002/cne.23009 22121037

[B6] Bekku Y. Su W.-D. Hirakawa S. Fässler R. Ohtsuka A. Kang J. S. (2003). Molecular Cloning of Bral2, a Novel Brain-specific Link Protein, and Immunohistochemical Colocalization with Brevican in Perineuronal Nets☆. Mol. Cell Neurosci. 24, 148–159. 10.1016/s1044-7431(03)00133-7 14550776

[B7] Bernard C. Prochiantz A. (2016). Otx2-PNN Interaction to Regulate Cortical Plasticity. Neural Plast. 2016, 7931693. 10.1155/2016/7931693 26881132 PMC4736602

[B8] Beurdeley M. Spatazza J. Lee H. H. C. Sugiyama S. Bernard C. di Nardo A. A. (2012). Otx2 Binding to Perineuronal Nets Persistently Regulates Plasticity in the Mature Visual Cortex. J. Neurosci. 32, 9429–9437. 10.1523/jneurosci.0394-12.2012 22764251 PMC3419577

[B9] Bosiacki M. Gąssowska-Dobrowolska M. Kojder K. Fabiańska M. Jeżewski D. Gutowska I. (2019). Perineuronal Nets and Their Role in Synaptic Homeostasis. Int. J. Mol. Sci. 20, 4108. 10.3390/ijms20174108 PMC6747153 31443560

[B10] Bozzelli P. L. Alaiyed S. Kim E. Villapol S. Conant K. (2018). Proteolytic Remodeling of Perineuronal Nets: Effects on Synaptic Plasticity and Neuronal Population Dynamics. Neural Plast. 2018, 5735789. 10.1155/2018/5735789 29531525 PMC5817213

[B11] Brakebusch C. Seidenbecher C. I. Asztely F. Rauch U. Matthies H. Meyer H. (2002). Brevican-deficient Mice Display Impaired Hippocampal CA1 Long-Term Potentiation but Show No Obvious Deficits in Learning and Memory. Mol. Cel Biol 22, 7417–7427. 10.1128/mcb.22.21.7417-7427.2002 PMC135663 12370289

[B12] Brückner G. Grosche J. Schmidt S. Härtig W. Margolis R. U. Delpech B. (2000). Postnatal Development of Perineuronal Nets in Wild-type Mice and in a Mutant Deficient in Tenascin-R. J. Comp. Neurol. 428, 616–629. 10.1002/1096-9861(20001225)428:4<616::aid-cne3>3.0.co;2-k 11077416

[B13] Brückner G. Grosche J. Hartlage-Rübsamen M. Schmidt S. Schachner M. (2003). Region and Lamina-specific Distribution of Extracellular Matrix Proteoglycans, Hyaluronan and Tenascin-R in the Mouse Hippocampal Formation. J. Chem. Neuroanat. 26, 37–50. 10.1016/s0891-0618(03)00036-x 12954529

[B14] Brückner G. Szeöke S. Pavlica S. Grosche J. Kacza J. (2006). Axon Initial Segment Ensheathed by Extracellular Matrix in Perineuronal Nets. Neuroscience 138, 365–375. 10.1016/j.neuroscience.2005.11.068 16427210

[B15] Bukalo O. Schachner M. Dityatev A. (2001). Modification of Extracellular Matrix by Enzymatic Removal of Chondroitin Sulfate and by Lack of Tenascin-R Differentially Affects Several Forms of Synaptic Plasticity in the hippocampus. Neuroscience 104, 359–369. 10.1016/s0306-4522(01)00082-3 11377840

[B16] Cabungcal J.-H. Steullet P. Morishita H. Kraftsik R. Cuenod M. Hensch T. K. (2013). Perineuronal Nets Protect Fast-Spiking Interneurons against Oxidative Stress. Proc. Natl. Acad. Sci. U.S.A. 110, 9130–9135. 10.1073/pnas.1300454110 23671099 PMC3670388

[B17] Carstens K. E. Phillips M. L. Pozzo-Miller L. Weinberg R. J. Dudek S. M. (2016). Perineuronal Nets Suppress Plasticity of Excitatory Synapses on CA2 Pyramidal Neurons. J. Neurosci. 36, 6312–6320. 10.1523/jneurosci.0245-16.2016 27277807 PMC4899529

[B18] Celio M. R. Spreafico R. de Biasi S. Vitellaro-Zuccarello L. (1998). Perineuronal Nets: Past and Present. Trends Neurosciences 21, 510–515. 10.1016/s0166-2236(98)01298-3 9881847

[B19] Corvetti L. Rossi F. (2005). Degradation of Chondroitin Sulfate Proteoglycans Induces Sprouting of Intact Purkinje Axons in the Cerebellum of the Adult Rat. J. Neurosci. 25, 7150–7158. 10.1523/jneurosci.0683-05.2005 16079397 PMC6725229

[B20] Deepa S. S. Carulli D. Galtrey C. Rhodes K. Fukuda J. Mikami T. (2006). Composition of Perineuronal Net Extracellular Matrix in Rat Brain. J. Biol. Chem. 281, 17789–17800. 10.1074/jbc.m600544200 16644727

[B21] Devienne G. Picaud S. Cohen I. Piquet J. Tricoire L. Testa D. (2021). Regulation of Perineuronal Nets in the Adult Cortex by the Activity of the Cortical Network. J. Neurosci. 41 (27), 5779–5790. 10.1523/jneurosci.0434-21.2021 PMC8265812 34045309

[B22] Dityatev A. Brückner G. Dityateva G. Grosche J. Kleene R. Schachner M. (2007). Activity-dependent Formation and Functions of Chondroitin Sulfate-Rich Extracellular Matrix of Perineuronal Nets. Devel Neurobio 67, 570–588. 10.1002/dneu.20361 17443809

[B23] Dzyubenko E. Fleischer M. Manrique-Castano D. Borbor M. Kleinschnitz C. Faissner A. (2021). Inhibitory Control in Neuronal Networks Relies on the Extracellular Matrix Integrity. Cell Mol Life Sci 78, 5647–5663. 10.1007/s00018-021-03861-3 34128077 PMC8257544

[B24] Dzyubenko E. Gottschling C. Faissner A. (2016). Neuron-Glia Interactions in Neural Plasticity: Contributions of Neural Extracellular Matrix and Perineuronal Nets. Neural Plast. 2016, 5214961. 10.1155/2016/5214961 26881114 PMC4736403

[B25] Edelstein L. Smythies J. (2013). Hypotheses Concerning How Otx2 Makes its Incredible Journey: a Hitchhiker on the Road to Rome? Front. Mol. Neurosci. 6, 55. 10.3389/fnmol.2013.00055 24415998 PMC3874477

[B26] Faini G. Aguirre A. Landi S. Lamers D. Pizzorusso T. Ratto G. M. (2018). Perineuronal Nets Control Visual Input via Thalamic Recruitment of Cortical PV Interneurons. Elife 7. 10.7554/eLife.41520 PMC6298774 30561327

[B97] Faissner A. Kruse J. (1990). J1/tenascin is a Repulsive Substrate for Central Nervous System Neurons. Neuron 5, 627–637. 1699568 10.1016/0896-6273(90)90217-4

[B27] Faissner A. Pyka M. Geissler M. Sobik T. Frischknecht R. Gundelfinger E. D. (2010). Contributions of Astrocytes to Synapse Formation and Maturation - Potential Functions of the Perisynaptic Extracellular Matrix. Brain Res. Rev. 63, 26–38. 10.1016/j.brainresrev.2010.01.001 20096729

[B28] Favuzzi E. Marques-Smith A. Deogracias R. Winterflood C. M. Sánchez-Aguilera A. Mantoan L. (2017). Activity-Dependent Gating of Parvalbumin Interneuron Function by the Perineuronal Net Protein Brevican. Neuron 95, 639–655. 10.1016/j.neuron.2017.06.028 28712654

[B29] Fawcett J. W. Oohashi T. Pizzorusso T. (2019). The Roles of Perineuronal Nets and the Perinodal Extracellular Matrix in Neuronal Function. Nat. Rev. Neurosci. 20, 451–465. 10.1038/s41583-019-0196-3 31263252

[B30] Fogarty M. J. Hammond L. A. Kanjhan R. Bellingham M. C. Noakes P. G. (2013). A Method for the Three-Dimensional Reconstruction of Neurobiotin-Filled Neurons and the Location of Their Synaptic Inputs. Front. Neural Circuits 7, 153. 10.3389/fncir.2013.00153 24101895 PMC3787200

[B31] Forsberg E. Hirsch E. Fröhlich L. Meyer M. Ekblom P. Aszodi A. (1996). Skin Wounds and Severed Nerves Heal Normally in Mice Lacking Tenascin-C. Proc. Natl. Acad. Sci. U.S.A. 93, 6594–6599. 10.1073/pnas.93.13.6594 8692862 PMC39070

[B32] Frantz C. Stewart K. M. Weaver V. M. (2010). The Extracellular Matrix at a Glance. J. Cel Sci 123, 4195–4200. 10.1242/jcs.023820 PMC2995612 21123617

[B33] Frischknecht R. Gundelfinger E. D. (2012). The Brain's Extracellular Matrix and its Role in Synaptic Plasticity. Adv. Exp. Med. Biol. 970, 153–171. 10.1007/978-3-7091-0932-8_7 22351055

[B34] Galtrey C. M. Kwok J. C. F. Carulli D. Rhodes K. E. Fawcett J. W. (2008). Distribution and Synthesis of Extracellular Matrix Proteoglycans, Hyaluronan, Link Proteins and Tenascin-R in the Rat Spinal Cord. Eur. J. Neurosci. 27, 1373–1390. 10.1111/j.1460-9568.2008.06108.x 18364019

[B35] Geissler M. Gottschling C. Aguado A. Rauch U. Wetzel C. H. Hatt H. (2013). Primary Hippocampal Neurons, Which Lack Four Crucial Extracellular Matrix Molecules, Display Abnormalities of Synaptic Structure and Function and Severe Deficits in Perineuronal Net Formation. J. Neurosci. 33, 7742–7755. 10.1523/jneurosci.3275-12.2013 23637166 PMC6618965

[B36] Giamanco K. A. Morawski M. Matthews R. T. (2010). Perineuronal Net Formation and Structure in Aggrecan Knockout Mice. Neuroscience 170, 1314–1327. 10.1016/j.neuroscience.2010.08.032 20732394

[B37] Gottschling C. Wegrzyn D. Denecke B. Faissner A. (2019). Elimination of the Four Extracellular Matrix Molecules Tenascin-C, Tenascin-R, Brevican and Neurocan Alters the Ratio of Excitatory and Inhibitory Synapses. Sci. Rep. 9, 13939. 10.1038/s41598-019-50404-9 31558805 PMC6763627

[B38] Happel M. F. Frischknecht R. (2016). Neuronal Plasticity in the Juvenile and Adult Brain Regulated by the Extracellular Matrix. London: IntechOpen.

[B39] Härtig W. Brauer K. Brückner G. (1992). Wisteria Floribunda Agglutinin-Labelled Nets Surround Parvalbumin-Containing Neurons. Neuroreport 3, 869–872. 10.1097/00001756-199210000-00012 1421090

[B40] Herrera F. Sainz R. M. Mayo J. C. Martín V. Antolín I. Rodriguez C. (2001). Glutamate Induces Oxidative Stress Not Mediated by Glutamate Receptors or Cystine Transporters: Protective Effect of Melatonin and Other Antioxidants. J. Pineal Res. 31, 356–362. 10.1034/j.1600-079x.2001.310411.x 11703566

[B41] Hunt C. Schenker L. Kennedy M. (1996). PSD-95 Is Associated with the Postsynaptic Density and Not with the Presynaptic Membrane at Forebrain Synapses. J. Neurosci. 16, 1380–1388. 10.1523/jneurosci.16-04-01380.1996 8778289 PMC6578559

[B42] Hunyadi A. Gaál B. Matesz C. Meszar Z. Morawski M. Reimann K. (2020). Distribution and Classification of the Extracellular Matrix in the Olfactory Bulb. Brain Struct. Funct. 225, 321–344. 10.1007/s00429-019-02010-8 31858237 PMC6957564

[B43] Hynes R. O. Naba A. (2012). Overview of the Matrisome-Aan Inventory of Extracellular Matrix Constituents and Functions. Cold Spring Harbor Perspect. Biol. 4, a004903. 10.1101/cshperspect.a004903 PMC3249625 21937732

[B44] Hynes R. O. (2009). The Extracellular Matrix: Not Just Pretty Fibrils. Science 326, 1216–1219. 10.1126/science.1176009 19965464 PMC3536535

[B45] Irintchev A. Rollenhagen A. Troncoso E. Kiss J. Z. Schachner M. (2005). Structural and Functional Aberrations in the Cerebral Cortex of Tenascin-C Deficient Mice. Cereb. Cortex 15, 950–962. 10.1093/cercor/bhh195 15537675

[B46] Jakovljevic A. Tucic M. Blazikova M. Korenic A. Missirlis Y. Stamenkovic V. (2021). Structural and Functional Modulation of Perineuronal Nets: In Search of Important Players with Highlight on Tenascins. Cells 10, 1345. 10.3390/cells10061345 34072323 PMC8230358

[B47] Jansen S. Gottschling C. Faissner A. Manahan-Vaughan D. (2017). Intrinsic Cellular and Molecular Properties of In Vivo Hippocampal Synaptic Plasticity Are Altered in the Absence of Key Synaptic Matrix Molecules. Hippocampus 27, 920–933. 10.1002/hipo.22742 28512860

[B48] Lee E. Lee J. Kim E. (2017). Excitation/Inhibition Imbalance in Animal Models of Autism Spectrum Disorders. Biol. Psychiatry 81, 838–847. 10.1016/j.biopsych.2016.05.011 27450033

[B49] Lee H. H. C. Bernard C. Ye Z. Acampora D. Simeone A. Prochiantz A. (2017). Genetic Otx2 Mis-Localization Delays Critical Period Plasticity across Brain Regions. Mol. Psychiatry 22, 680–688. 10.1038/mp.2017.1 28194008 PMC5400722

[B50] Lemarchant S. Wojciechowski S. Koistinaho J. (2016). Perineuronal Nets in Neurodegeneration. Oncotarget 7, 78224–78225. 10.18632/oncotarget.13420 27861161 PMC5346633

[B51] Lensjø K. K. Christensen A. C. Tennøe S. Fyhn M. Hafting T. (2017). Differential Expression and Cell-type Specificity of Perineuronal Nets in Hippocampus, Medial Entorhinal Cortex, and Visual Cortex Examined in the Rat and Mouse. eNeuro 4, ENEURO.0379-16.2017. 10.1523/ENEURO.0379-16.2017 PMC5461557 28593193

[B52] Levelt C. N. Hübener M. (2012). Critical-period Plasticity in the Visual Cortex. Annu. Rev. Neurosci. 35, 309–330. 10.1146/annurev-neuro-061010-113813 22462544

[B53] Lu J. Tucciarone J. Lin Y. Huang Z. J. (2014). Input-specific Maturation of Synaptic Dynamics of Parvalbumin Interneurons in Primary Visual Cortex. Proc. Natl. Acad. Sci. U.S.A. 111, 16895–16900. 10.1073/pnas.1400694111 25385583 PMC4250102

[B54] Lundell A. Olin A. I. Mörgelin M. al-Karadaghi S. Aspberg A. Logan D. T. (2004). Structural Basis for Interactions between Tenascins and Lectican C-type Lectin Domains. Structure 12, 1495–1506. 10.1016/j.str.2004.05.021 15296743

[B55] Maeda N. (2015). Proteoglycans and Neuronal Migration in the Cerebral Cortex during Development and Disease. Front. Neurosci. 9, 98. 10.3389/fnins.2015.00098 25852466 PMC4369650

[B56] Matthews R. T. Kelly G. M. Zerillo C. A. Gray G. Tiemeyer M. Hockfield S. (2002). Aggrecan Glycoforms Contribute to the Molecular Heterogeneity of Perineuronal Nets. J. Neurosci. 22, 7536–7547. 10.1523/jneurosci.22-17-07536.2002 12196577 PMC6757962

[B57] Miyata S. Nadanaka S. Igarashi M. Kitagawa H. (2018). Structural Variation of Chondroitin Sulfate Chains Contributes to the Molecular Heterogeneity of Perineuronal Nets. Front. Integr. Neurosci. 12, 3. 10.3389/fnint.2018.00003 29456495 PMC5801575

[B58] Mjaatvedt C. H. Yamamura H. Capehart A. A. Turner D. Markwald R. R. (1998). TheCspg2Gene, Disrupted in thehdfMutant, Is Required for Right Cardiac Chamber and Endocardial Cushion Formation. Develop. Biol. 202, 56–66. 10.1006/dbio.1998.9001 9758703

[B98] Mueller-Buehl C. Reinhard J. Roll L. Bader V. Winklhofer K.F. Faissner A. (2022). Brevican, Neurocan, Tenascin-C and Tenascin-R Act as Important Regulators of the Interplay between Perineuronal Nets, Synaptic Integrity, Inhibitory Interneurons and Otx2. bioRxiv. 10.1101/2022.02.24.481837 PMC9201762 35721494

[B59] Morawski M. Dityatev A. Hartlage-Rübsamen M. Blosa M. Holzer M. Flach K. (2014). Tenascin-R Promotes Assembly of the Extracellular Matrix of Perineuronal Nets via Clustering of Aggrecan. Phil. Trans. R. Soc. B 369, 20140046. 10.1098/rstb.2014.0046 25225104 PMC4173296

[B60] Murphy S. Rudge J. (1985). Glycoprotein Composition and Turnover in Subcellular Fractions from the Cerebral Cortex of normal and Reeler Mutant Mice. Develop. Brain Res. 21, 73–81. 10.1016/0165-3806(85)90024-0 4027684

[B61] Nadanaka S. Miyata S. Yaqiang B. Tamura J. I. Habuchi O. Kitagawa H. (2020). Reconsideration of the Semaphorin-3A Binding Motif Found in Chondroitin Sulfate Using Galnac4s-6st-Knockout Mice. Biomolecules 10, 1499. 10.3390/biom10111499 PMC7694144 33143303

[B62] Naito S. Ueda T. (1985). Characterization of Glutamate Uptake into Synaptic Vesicles. J. Neurochem. 44, 99–109. 10.1111/j.1471-4159.1985.tb07118.x 2856886

[B63] Nelson S. B. Valakh V. (2015). Excitatory/Inhibitory Balance and Circuit Homeostasis in Autism Spectrum Disorders. Neuron 87, 684–698. 10.1016/j.neuron.2015.07.033 26291155 PMC4567857

[B64] Padhi A. Nain A. S. (2020). ECM in Differentiation: A Review of Matrix Structure, Composition and Mechanical Properties. Ann. Biomed. Eng. 48, 1071–1089. 10.1007/s10439-019-02337-7 31485876

[B99] Pfaffl M. W. Horgan G. W. Dempfle L. (2002). Relative Expression Software Tool (REST) for Group-Wise Comparison and Statistical Analysis of Relative Expression Results in Real-Time PCR. Nucl. Acids Res. 30, e36. 11972351 10.1093/nar/30.9.e36 PMC113859

[B65] Pizzorusso T. Medini P. Berardi N. Chierzi S. Fawcett J. W. Maffei L. (2002). Reactivation of Ocular Dominance Plasticity in the Adult Visual Cortex. Science 298, 1248–1251. 10.1126/science.1072699 12424383

[B66] Pizzorusso T. Medini P. Landi S. Baldini S. Berardi N. Maffei L. (2006). Structural and Functional Recovery from Early Monocular Deprivation in Adult Rats. Proc. Natl. Acad. Sci. U.S.A. 103, 8517–8522. 10.1073/pnas.0602657103 16709670 PMC1482523

[B67] Planques A. Oliveira Moreira V. Benacom D. Bernard C. Jourdren L. Blugeon C. (2021). OTX2 Homeoprotein Functions in Adult Choroid Plexus. Int. J. Mol. Sci. 22, 8951. 10.3390/ijms22168951 34445655 PMC8396604

[B68] Planques A. Oliveira Moreira V. Dubreuil C. Prochiantz A. di Nardo A. A. (2019). OTX2 Signals from the Choroid Plexus to Regulate Adult Neurogenesis. eNeuro 6, ENEURO.0262-18.2019. 10.1523/ENEURO.0262-18.2019 PMC6506823 31064838

[B69] Pyka M. Wetzel C. Aguado A. Geissler M. Hatt H. Faissner A. (2011). Chondroitin Sulfate Proteoglycans Regulate Astrocyte-dependent Synaptogenesis and Modulate Synaptic Activity in Primary Embryonic Hippocampal Neurons. Eur. J. Neurosci. 33, 2187–2202. 10.1111/j.1460-9568.2011.07690.x 21615557

[B100] Rathjen F. G. Wolff J. M. Chiquet-Ehrismann R. (1991). Restrictin: A Chick Neural Extracellular Matrix Protein Involved in Cell Attachment Co-Purifies With the Cell Recognition Molecule F11. Development 113, 151–164. 1764992 10.1242/dev.113.1.151

[B70] Rauch U. Zhou X.-H. Roos G. (2005). Extracellular Matrix Alterations in Brains Lacking Four of its Components. Biochem. Biophysical Res. Commun. 328, 608–617. 10.1016/j.bbrc.2005.01.026 15694392

[B71] Reichelt A. C. Hare D. J. Bussey T. J. Saksida L. M. (2019). Perineuronal Nets: Plasticity, Protection, and Therapeutic Potential. Trends Neurosciences 42, 458–470. 10.1016/j.tins.2019.04.003 31174916

[B73] Reinehr S. Reinhard J. Wiemann S. Stute G. Kuehn S. Woestmann J. (2016). Early Remodelling of the Extracellular Matrix Proteins Tenascin-C and Phosphacan in Retina and Optic Nerve of an Experimental Autoimmune Glaucoma Model. J. Cel. Mol. Med. 20, 2122–2137. 10.1111/jcmm.12909 PMC5082392 27374750

[B74] Rowlands D. Lensjø K. K. Dinh T. Yang S. Andrews M. R. Hafting T. (2018). Aggrecan Directs Extracellular Matrix-Mediated Neuronal Plasticity. J. Neurosci. 38, 10102–10113. 10.1523/jneurosci.1122-18.2018 30282728 PMC6596198

[B75] Sakai A. Nakato R. Ling Y. Hou X. Hara N. Iijima T. (2017). Genome-Wide Target Analyses of Otx2 Homeoprotein in Postnatal Cortex. Front. Neurosci. 11, 307. 10.3389/fnins.2017.00307 28620275 PMC5450002

[B76] Sala C. Vicidomini C. Bigi I. Mossa A. Verpelli C. (2015). Shank Synaptic Scaffold Proteins: Keys to Understanding the Pathogenesis of Autism and Other Synaptic Disorders. J. Neurochem. 135, 849–858. 10.1111/jnc.13232 26338675

[B77] Saroja S. R. Sase A. Kircher S. G. Wan J. Berger J. Höger H. (2014). Hippocampal Proteoglycans Brevican and Versican Are Linked to Spatial Memory of Sprague-Dawley Rats in the morris Water Maze. J. Neurochem. 130, 797–804. 10.1111/jnc.12783 24903590

[B78] Schätzle P. Wuttke R. Ziegler U. Sonderegger P. (2012). Automated Quantification of Synapses by Fluorescence Microscopy. J. Neurosci. Methods 204, 144–149. 10.1016/j.jneumeth.2011.11.010 22108140

[B79] Sigal Y. M. Bae H. Bogart L. J. Hensch T. K. Zhuang X. (2019). Structural Maturation of Cortical Perineuronal Nets and Their Perforating Synapses Revealed by Superresolution Imaging. Proc. Natl. Acad. Sci. U.S.A. 116, 7071–7076. 10.1073/pnas.1817222116 30890637 PMC6452715

[B80] Sohal V. S. Rubenstein J. L. R. (2019). Excitation-inhibition Balance as a Framework for Investigating Mechanisms in Neuropsychiatric Disorders. Mol. Psychiatry 24, 1248–1257. 10.1038/s41380-019-0426-0 31089192 PMC6742424

[B81] Spatazza J. Lee H. H. C. Di Nardo A. A. Tibaldi L. Joliot A. Hensch T. K. (2013). Choroid-plexus-derived Otx2 Homeoprotein Constrains Adult Cortical Plasticity. Cel Rep. 3, 1815–1823. 10.1016/j.celrep.2013.05.014 PMC4119931 23770240

[B82] Stamenkovic V. Stamenkovic S. Jaworski T. Gawlak M. Jovanovic M. Jakovcevski I. (2017). The Extracellular Matrix Glycoprotein Tenascin-C and Matrix Metalloproteinases Modify Cerebellar Structural Plasticity by Exposure to an Enriched Environment. Brain Struct. Funct. 222, 393–415. 10.1007/s00429-016-1224-y 27089885

[B101] Stirling D. R. Swain-Bowden M. J. Lucas A. M. Carpenter A. E. Cimini B. A Goodmann A. (2021). CellProfiler 4: Improvements in Speed, Utility and Usability. BMC Bioinform. 22, 433. 10.1186/s12859-021-04344-9 PMC8431850 34507520

[B83] Sugiyama S. di Nardo A. A. Aizawa S. Matsuo I. Volovitch M. Prochiantz A. (2008). Experience-dependent Transfer of Otx2 Homeoprotein into the Visual Cortex Activates Postnatal Plasticity. Cell 134, 508–520. 10.1016/j.cell.2008.05.054 18692473

[B84] Suttkus A. Rohn S. Jäger C. Arendt T. Morawski M. (2012). Neuroprotection against Iron-Induced Cell Death by Perineuronal Nets - an In Vivo Analysis of Oxidative Stress. Am. J. Neurodegener Dis. 1, 122–129. 23383386 PMC3560462

[B85] Takesian A. E. Hensch T. K. (2013). Balancing Plasticity/stability across Brain Development. Prog. Brain Res. 207, 3–34. 10.1016/b978-0-444-63327-9.00001-1 24309249

[B86] Tamás G. Buhl E. H. Lörincz A. Somogyi P. (2000). Proximally Targeted GABAergic Synapses and gap Junctions Synchronize Cortical Interneurons. Nat. Neurosci. 3, 366–371. 10.1038/73936 10725926

[B87] van 't Spijker H. M. Kwok J. C. F. (2017). A Sweet Talk: The Molecular Systems of Perineuronal Nets in Controlling Neuronal Communication. Front. Integr. Neurosci. 11, 33. 10.3389/fnint.2017.00033 29249944 PMC5717013

[B88] Wang D. Fawcett J. (2012). The Perineuronal Net and the Control of CNS Plasticity. Cel Tissue Res 349, 147–160. 10.1007/s00441-012-1375-y 22437874

[B89] Wang W. Passaniti A. (1999). Extracellular Matrix Inhibits Apoptosis and Enhances Endothelial Cell Differentiation by a NfkappaB-dependent Mechanism. J. Cel. Biochem. 73, 321–331. 10.1002/(sici)1097-4644(19990601)73:3<321::aid-jcb4>3.0.co;2-0 10321832

[B90] Watanabe H. Kimata K. Line S. Strong D. Gao L.-y. Kozak C. A. (1994). Mouse Cartilage Matrix Deficiency (Cmd) Caused by a 7 Bp Deletion in the Aggrecan Gene. Nat. Genet. 7, 154–157. 10.1038/ng0694-154 7920633

[B91] Weber P. Bartsch U. Rasband M. N. Czaniera R. Lang Y. Bluethmann H. (1999). Mice Deficient for Tenascin-R Display Alterations of the Extracellular Matrix and Decreased Axonal Conduction Velocities in the CNS. J. Neurosci. 19, 4245–4262. 10.1523/jneurosci.19-11-04245.1999 10341229 PMC6782606

[B92] Xia D. Li L. Yang B. Zhou Q. (2021). Altered Relationship between Parvalbumin and Perineuronal Nets in an Autism Model. Front. Mol. Neurosci. 14, 597812. 10.3389/fnmol.2021.597812 33912009 PMC8072465

[B93] Yamaguchi Y. (2000). Lecticans: Organizers of the Brain Extracellular Matrix. Cmls, Cel. Mol. Life Sci. 57, 276–289. 10.1007/pl00000690 PMC11146776 10766023

[B94] Yue B. (2014). Biology of the Extracellular Matrix. J. Glaucoma 23, S20–S23. 10.1097/ijg.0000000000000108 25275899 PMC4185430

[B95] Zhou X.-H. Brakebusch C. Matthies H. Oohashi T. Hirsch E. Moser M. (2001). Neurocan Is Dispensable for Brain Development. Mol. Cel Biol 21, 5970–5978. 10.1128/mcb.21.17.5970-5978.2001 PMC87315 11486035

[B96] Zimmermann D. R. Dours-Zimmermann M. T. (2008). Extracellular Matrix of the central Nervous System: from Neglect to challenge. Histochem. Cel Biol 130, 635–653. 10.1007/s00418-008-0485-9 18696101

