# Supplementary material for: Brevican, Neurocan, Tenascin-C, and Tenascin-R Act as Important Regulators of the Interplay Between Perineuronal Nets, Synaptic Integrity, Inhibitory Interneurons, and Otx2
Source: Front Cell Dev Biol. 2022 Jun 2;10:886527. doi: 10.3389/fcell.2022.886527 (PMC9201762; doi:10.3389/fcell.2022.886527)

Supplementary Figure 1: Distribution of brevican, neurocan, tenascin-C and tenascin-R in the adult cerebral cortex. (A-D) Immunohistochemical stainings of brevican in adult murine coronal brain sections. Stainings revelead brevican-positive cells with a PNN-like structure (white arrows) in the RSC and V1 of adult wildtype mice. The antibody also showed unspecific stained blood vessels. In the V2M and Aud no cell attached brevican was detected. **(E-H)** Immunohistochemical stainings of neurocan in adult murine coronal brain sections. Stainings against neurocan exhibited positive cells in all regions. Immunoreactivity appeared to be especially strong in the V1 region. **(I-L)** Immunohistochemical stainings of tenascin-C in adult murine coronal brain sections. The tenascin-C staining could detect only unspecific background signal in all regions. Therefore, tenascin-C seems to be not present in the examined adult cortical areas. **(M-P)** Immunohistochemical stainings of tenascin-R in adult murine coronal brain sections. Tenascin-R stainings showed positive cells in RSC, V2M, V1, and Aud with an PNN like appearance (white arrows). But also, tenascin-R positive signal in the neuropil was detected by the staining. Aud = auditory cortex, V1 = primary visual cortex, V2 = secondary visual cortex, RSC = retrosplenial cortex, scale bars = 200 µm.

Supplementary Figure 2: Diminished PNN organization in the retrosplenial-, secondary visual- and auditory cortex of quadruple knockout mice. **(A-F)** Immunohistochemical staining of PNNs in murine coronal brain slices with WFA (green) and anti-aggrecan (red). Images of WFA-positive and aggrecan-positive PNN-enwrapped neurons were recorded and counted in the retrosplenial-, secondary visual- and auditory cortex. **(G)** A significantly reduced number of WFA-positive cells in the retrosplenial-, secondary visual- and auditory cortex of quadruple knockout mice could be noticed ($p < 0.001$, N = 7). **(H)** Also, the number of aggrecan-positive cells was significantly reduced in retrosplenial-, secondary visual- and auditory cortex of quadruple Knockout mice ($p < 0.001$, N = 7). **(I, J)** The number of WFA-positive and aggrecan-positive processes per PNN were counted in retrosplenial-, secondary visual-, primary visual- and auditory cortex. Both, WFA-positive and aggrecan-positive processes were significantly reduced in the examined cortical areas ($p < 0.001$, N = 7); 4xKO = quadruple knockout, Aud = auditory cortex, V1 = primary visual cortex, V2 = secondary visual cortex, RSC = retrosplenial cortex, WFA = Wisteria floribunda agglutinin, WT = wildtype, *** = $p < 0.001$ data are shown as mean ± SEM and SD, scale bars = 20 µm.

Supplementary Figure 3: Analyses of synaptic puncta and spatial proximity between PNNs and gephyrin. (A) Representative images of immunohistochemical stainings of PNNs and inhibitory synaptic elements. WFA is used as marker for PNNs (blue), antibodies against gephyrin (green) and VGAT (red) as markers for inhibitory postsynaptic elements and inhibitory presynaptic elements, respectively. **(B)** Immunopositive signal of gephyrin and VGAT was recognized as synaptic puncta and represented as spots by fitting about the estimated puncta size of 0,3 µm and reaching the default intensity threshold. Immunoreactive signals not fitting these parameters were ignored as background noise. Synaptic spots of gephyrin and VGAT within a radius of 1 µm of each other were defined as colocalized and illustrated as green transparent or red transparent spots (yellow arrows). Gephyrin and VGAT spots outside of this radius were defined as non-colocalized and illustrated in dark green or rather dark red (orange arrows). **(C-D)** Representative wildtype PNN (blue) with gephyrin-positive synaptic puncta. Signal outside the PNN was suppressed. The overview image creates the impression of overlapping WFA and gephyrin signal. A close-up image shows that the signals are not overlapping but gephyrin-positive signal occurs in the immediate surrounding of WFA-positive PNN components. VGAT = vesicular GABA transporter, WFA = Wisteria floribunda agglutinin, scale bars = 0.3 µm.

Supplementary Figure 4: Inhibitory synaptic elements in the V1 of quadruple knockout mice. **(A)** Western blot analysis of gephyrin protein levels in the V1. **(B)** No significant differences in the gephyrin protein band intensity were detectable in visual cortex tissue of wildtype and quadruple knockout mice ($p = 0.16$, $N = 8$). **(C)** RT-qPCR analyses revealed a comparable *Gephn* mRNA expression in the visual cortex of wildtype and quadruple knockout mice ($p = 0.07$, $N = 6$). **(D)** Western blot analysis of VGAT protein levels in the V1. **(E)** Comparable VGAT protein band intensity in visual cortex tissue of wildtype and quadruple knockout ($p = 0.14$, $N = 8$). **(F)** RT-qPCR analyses revealed a significant lower *Slc32a1(VGAT)* mRNA expression in the visual cortex of quadruple knockout mice ($p < 0.001$, $N = 6$); 4xKO = quadruple knockout, *Gephn = Gephyrin*, V1 = primary visual cortex, VGAT = vesicular GABA transporter, WT = wildtype, * = $p < 0.05$ data are shown as mean ± SEM and SD.

Supplementary Figure 5: Excitatory synaptic elements in the V1 of quadruple knockout mice. **(A)** Western blot analysis of PSD95 protein levels in the V1. **(B)** No significant differences in the PSD95 protein band intensity were detectable in visual cortex tissue of wildtype and quadruple knockout mice ($p = 0.85$, $N = 8$). **(C)** RT-qPCR analyses revealed a comparable *Dlg4* (PSD95) mRNA expression in the visual cortex of wildtype and quadruple knockout mice ($p = 0.10$, $N = 6$). **(D)** Western blot analysis of VGLUT1 protein levels in the V1. **(E)** Comparable VGLUT1 protein band intensity in visual cortex tissue of wildtype and quadruple knockout ($p = 0.46$, $N = 8$). **(F)** RT-qPCR analyses revealed comparable *Slc17a7 (VGLUT1)* mRNA expression in the visual cortex of wildtype and quadruple knockout mice ($p = 0.40$, $N = 6$); 4xKO = quadruple knockout, *Dlg4 = postsynaptic density protein 95*, PSD95 = postsynaptic density protein 95, Slc17a7 = vesicular glutamate transporter 1, V1 = primary visual cortex, VGLUT1 = vesicular glutamate transporter 1, WT = wildtype, * = $p < 0.05$ data are shown as mean ± SEM and SD.

Supplementary Figure 6: Analyses of parvalbumin-positive interneuron populations in the retrosplenial-, secondary visual- and auditory cortex of wildtype and quadruple knockout mice. **(A-F)** Representative coronal cortical brain slices of wildtype and quadruple knockout double-labeled using a specific antibody against parvalbumin and WFA. **(G)** The number of parvalbumin-positive cells was comparable in the retrosplenial cortex in wildtype and quadruple knockout mice ($p = 0.67$, $N = 8$). Furthermore, the number of parvalbumin-positive cells was comparable in the secondary visual cortex ($p = 0.11$, $N = 8$) and the auditory cortex of wildtype and quadruple knockout mice ($p = 0.61$, $N = 8$). 4xKO = quadruple knockout, *Pvalb* = parvalbumin, WT = wildtype, WFA = *Wisteria floribunda* agglutinin, * = $p < 0.05$, data are shown as mean ± SEM and SD, scale bars = 200 µm.

Supplementary Figure 7: Analyses of calretinin-positive interneuron populations in the retrosplenial-, secondary visual- and auditory cortex of wildtype and quadruple knockout mice. **(A-F)** Representative coronal cortical brain slices of wildtype and quadruple knockout double-labeled using a specific antibody against calretinin and WFA retrosplenial-, secondary visual- and auditory cortex of wildtype and quadruple knockout mice. **(G)** The number of calretinin-positive cells was comparable in the retrosplenial cortex between wildtype and quadruple knockout mice ($p = 0.63$, $N = 8$). Furthermore, the number of calretinin-positive cells was comparable in the secondary visual cortex ($p = 0.53$, $N = 8$) and the auditory cortex of wildtype and quadruple knockout mice ($p = 0.19$, $N = 8$). 4xKO = quadruple knockout, *Pvalb* = parvalbumin, WT = wildtype, WFA = *Wisteria floribunda* agglutinin, * = $p < 0.05$, data are shown as mean ± SEM and SD, scale bars = 200 µm.

Supplement Figure 8: Comparable Otx2 internalization mediated by PNNs in quadruple knockout and wildtype RSC and V1. **(A-C, F-H)** Representative images of an immunohistochemical double staining with Otx2 (green) and WFA (red) in the RSC of quadruple knockout and wildtype mice. White arrows indicate Otx2 and WFA double-positive cells. **(D, I)** WFA stained PNNs were automatically

recognized by the CellProfiler software. The green dotted area was recognized as WFA-positive cell, whereas purple dotted areas with insufficient signal were depraved. **(E, J)** Representation of WFA-positive cells recognized by the CellProfiler software. Intensities of WFA and Otx2 immunoreactivity were measured inside these areas. **(K)** Statistical evaluation of WFA intensity measurements revealed a significant reduction in the quadruple knockout RSC ($p < 0.05$, N = 8) and V1 ($p < 0.005$, N = 8) in comparison to the wildtype. **(L)** In contrast, intensity measurements of Otx2 within the PNNs appeared to be comparable between wildtype and quadruple knockout in RSC ($p = 0.56$, N = 8) and V1 ($p = 0.38$, N = 8), indicating no impaired internalization of Otx2 through the disruption of the PNNs in the quadruple knockout. 4xKO = quadruple knockout, Otx2 = orthodenticle homeobox 2, RSC = retrosplenial cortex, V1 = primary visual cortex, WFA = Wisteria floribunda agglutinin, WT = wildtype, ** = $p < 0.005$, * = $p < 0.05$ data are shown as mean ± standard error mean and standard deviation, scale bars A = 20 µm.

17 SUPPLEMENTARY FIGURES

Supplementary Figure 1:

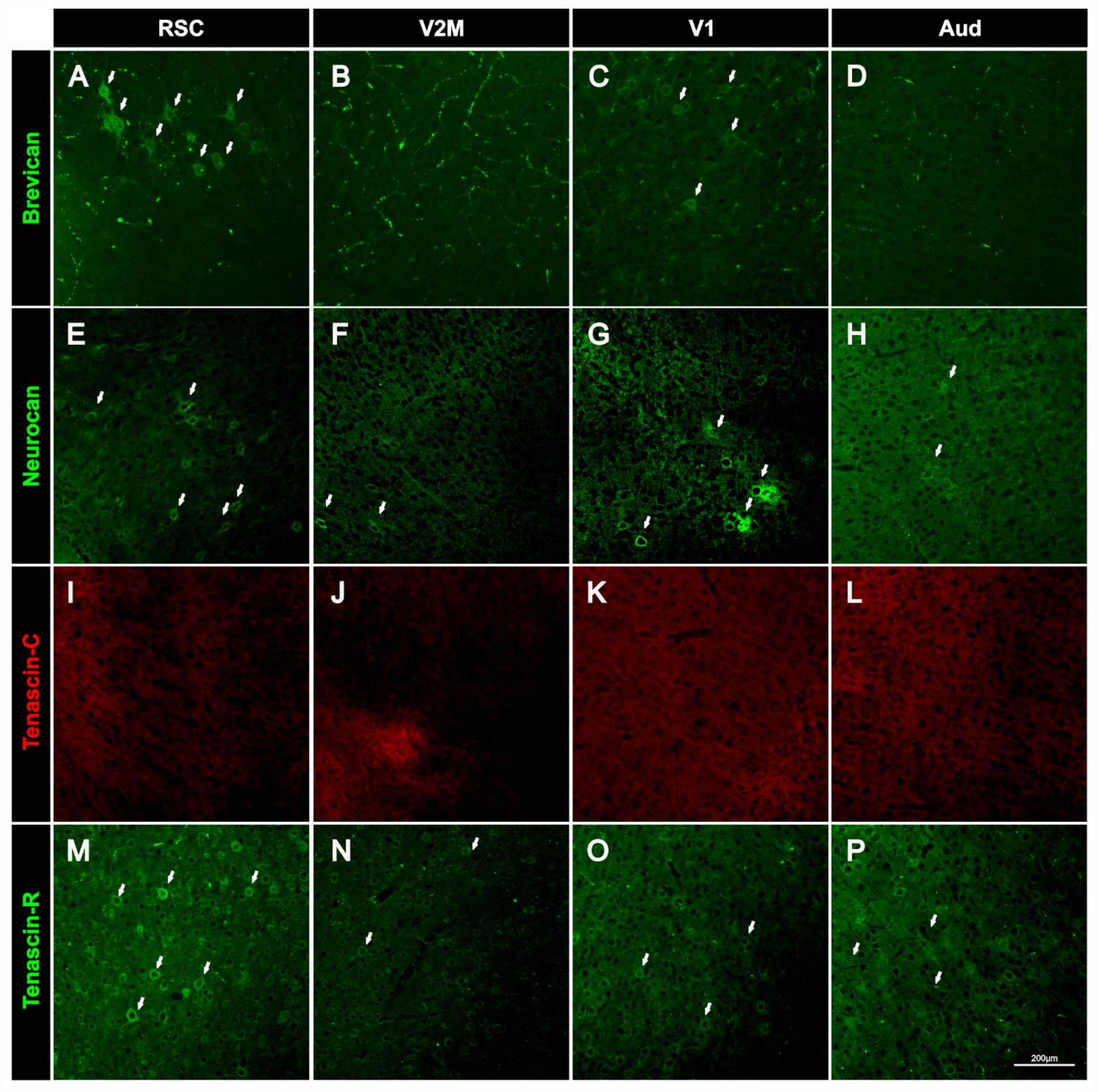

Supplementary Figure 2:

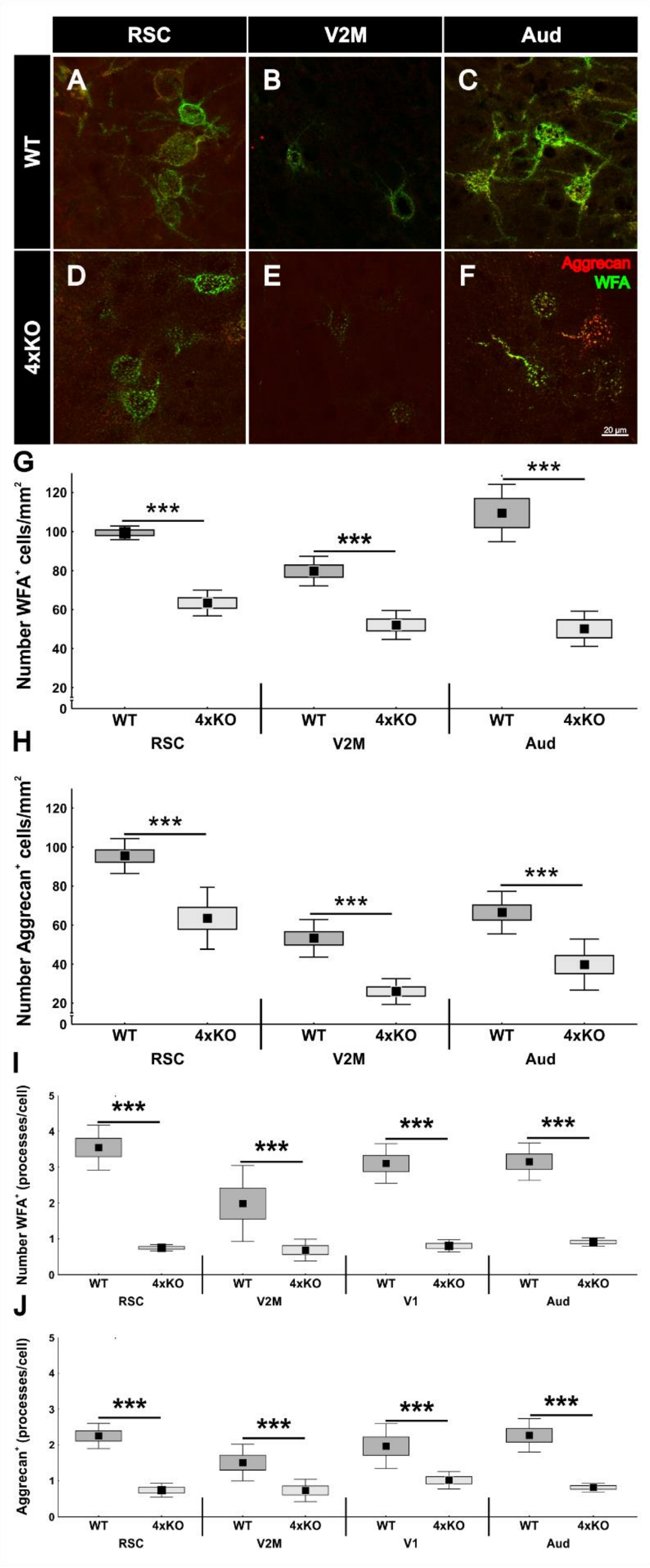

Supplementary Figure 3:

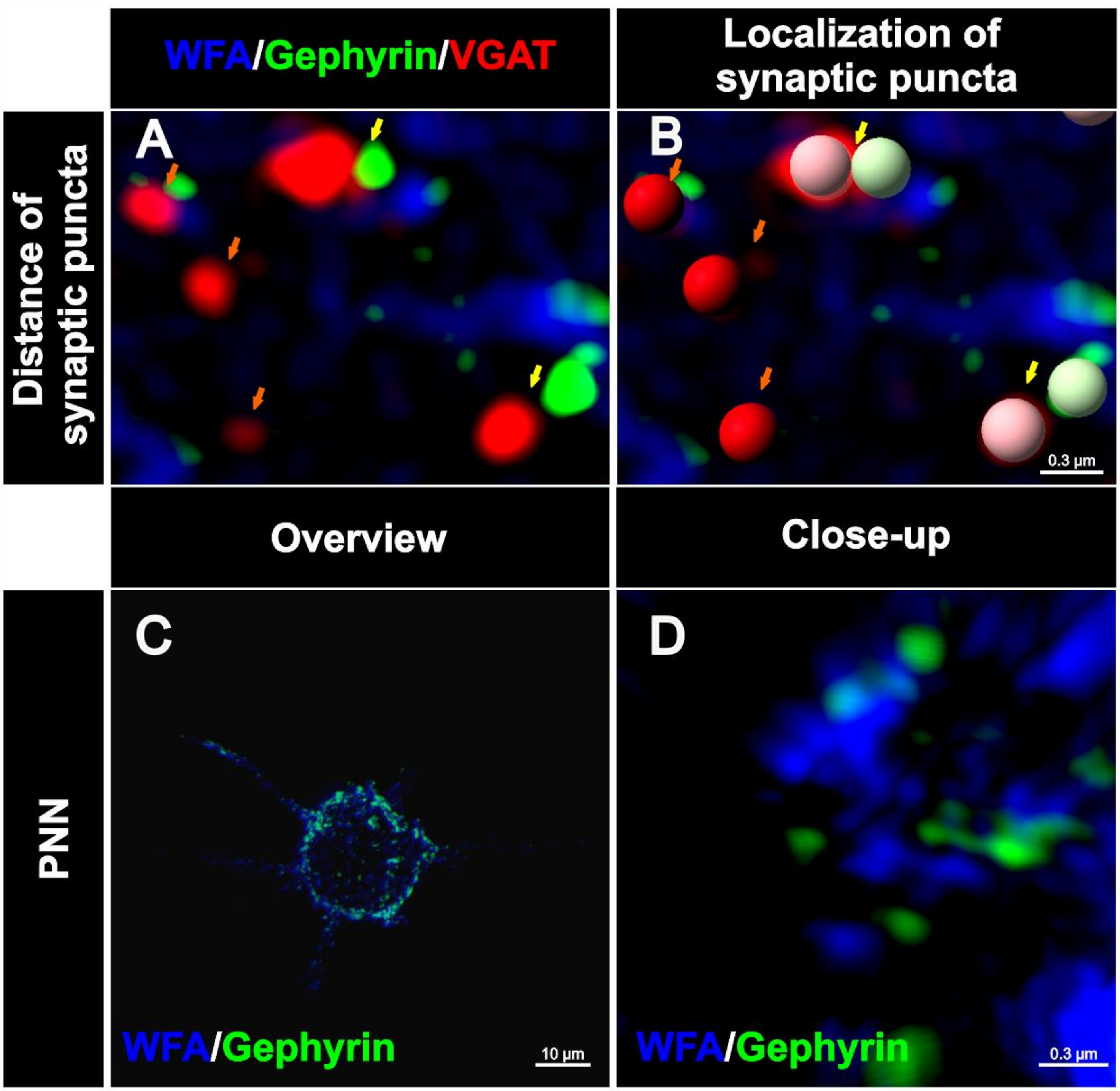

Supplementary Figure 4:

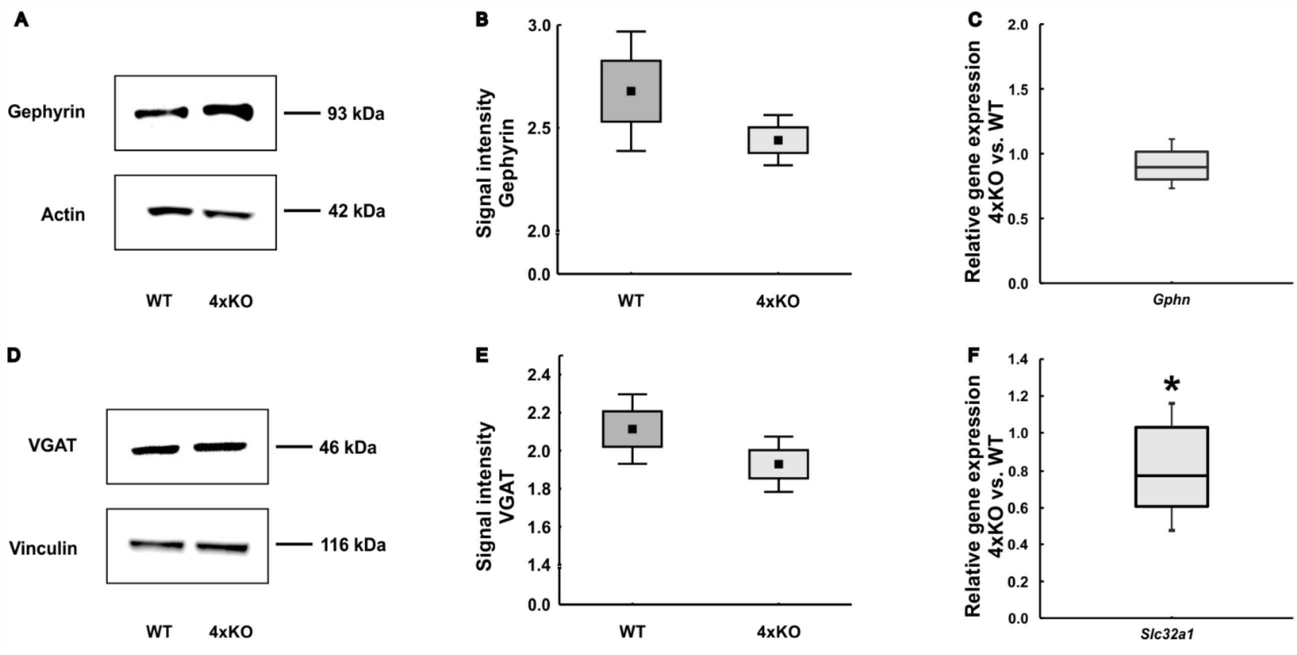

Supplementary Figure 5:

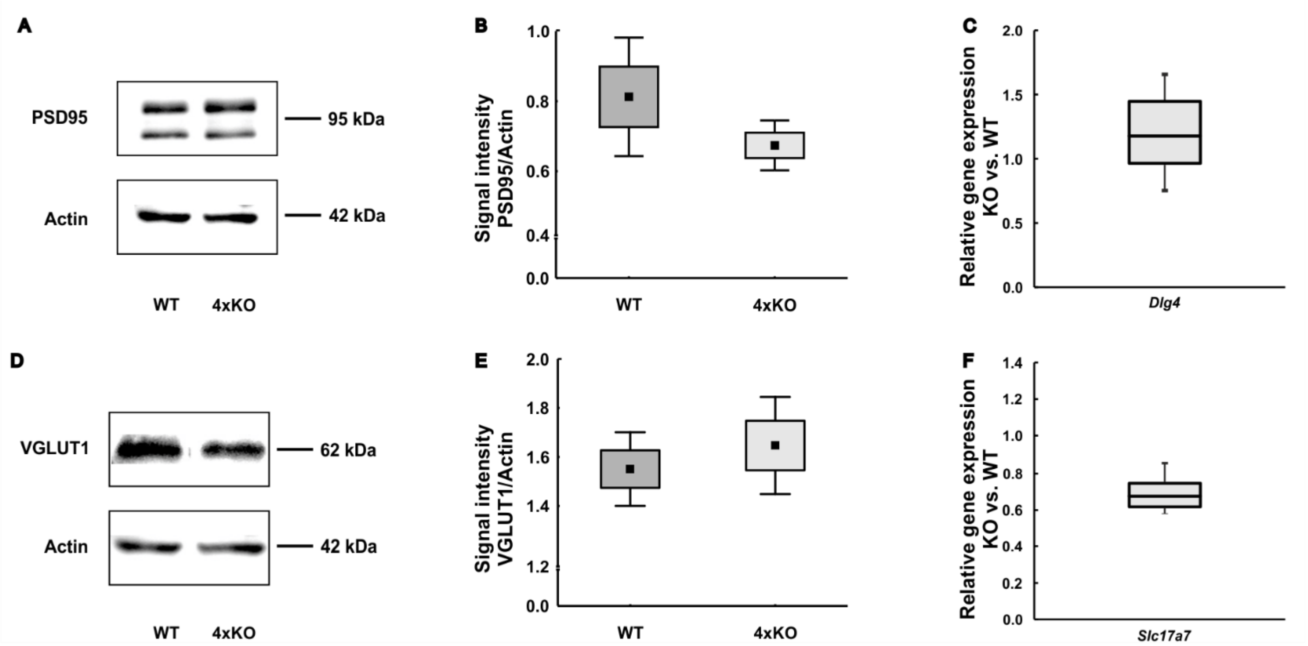

Supplementary Figure 6:

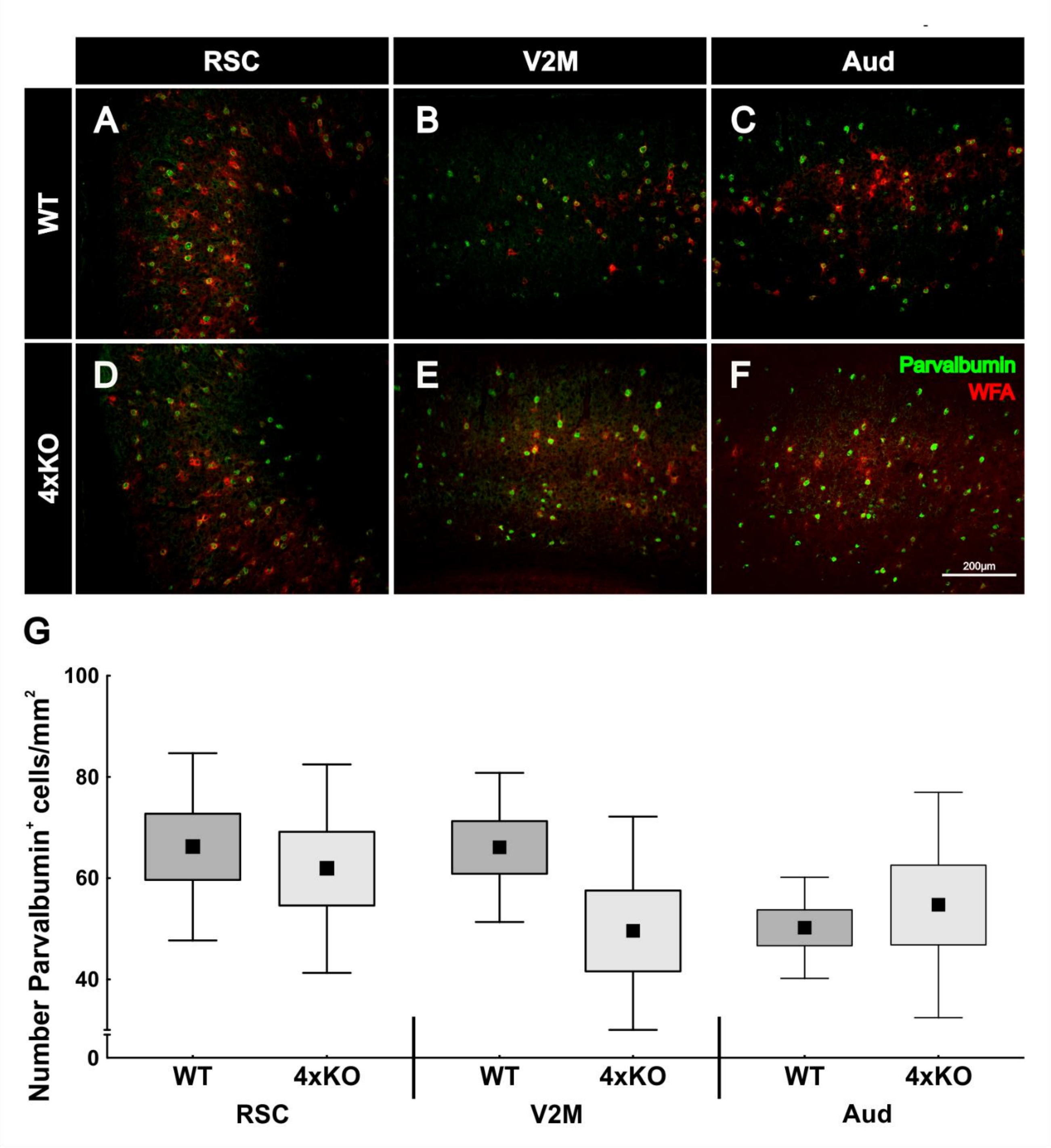

Supplementary Figure 7:

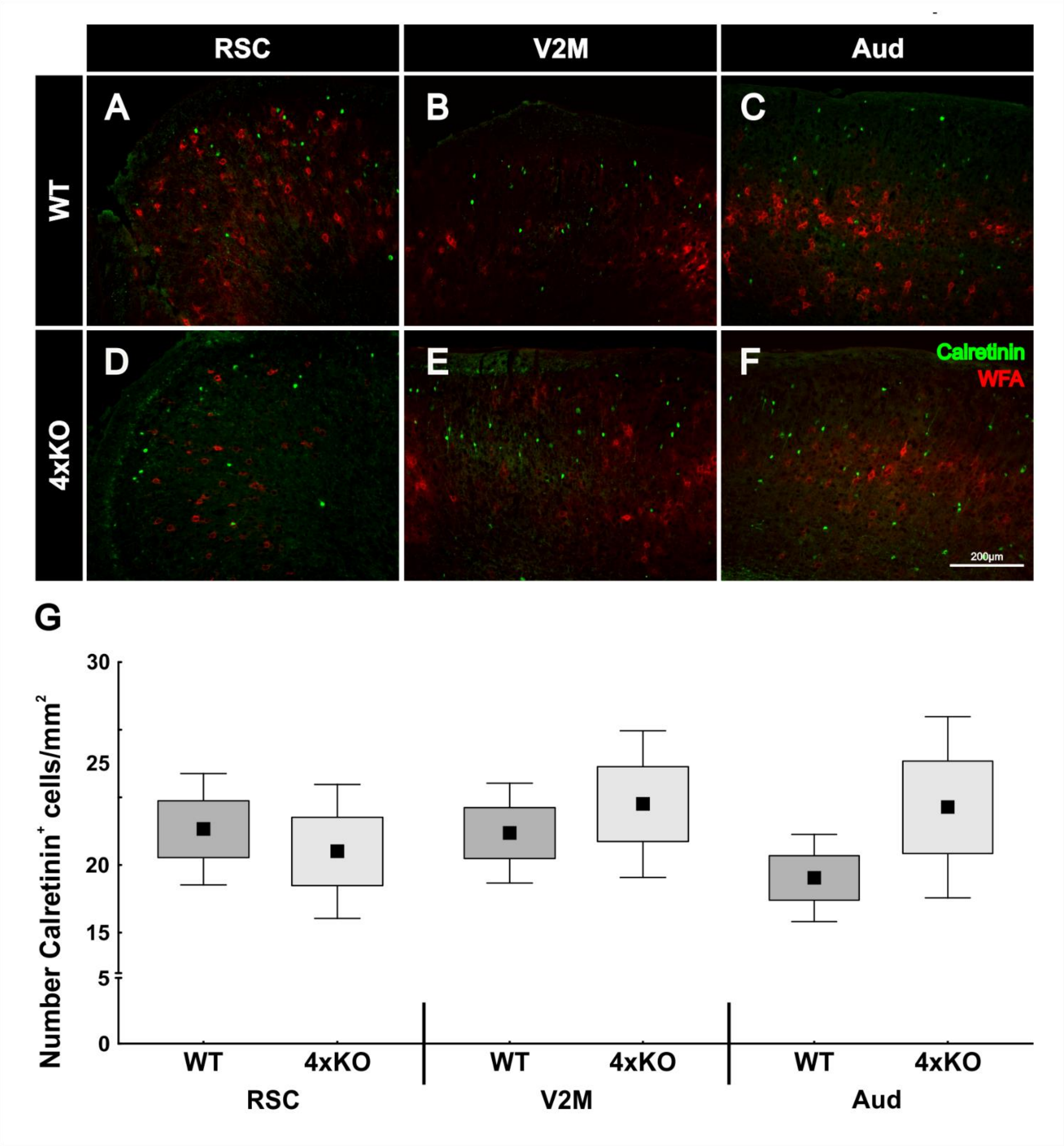

Supplementary Figure 8:

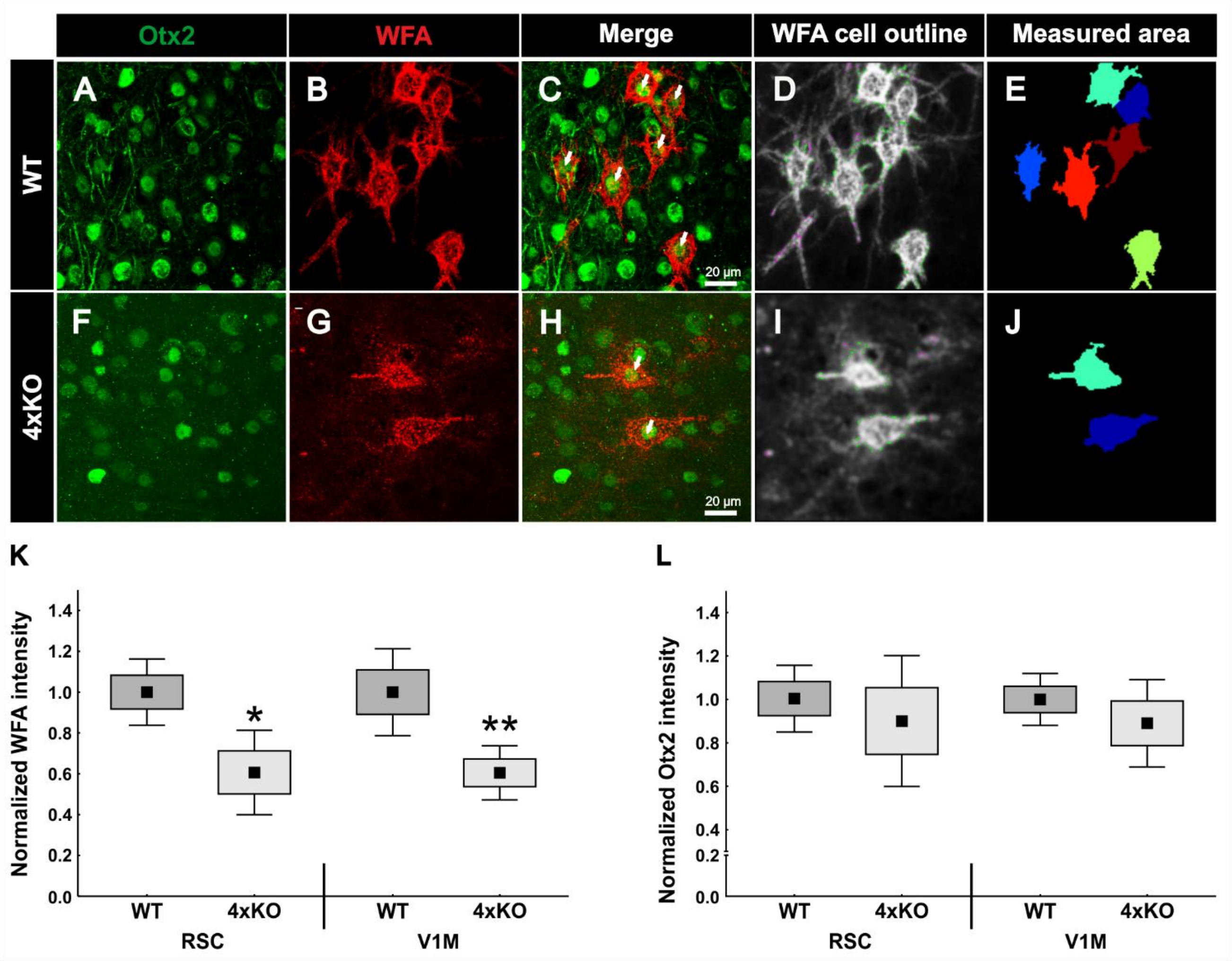

Supplement: Supplementary file 2 [file DataSheet1.pdf]
